# Targeted dephosphorylation of TFEB promotes its nuclear translocation

**DOI:** 10.1016/j.isci.2024.110432

**Published:** 2024-06-29

**Authors:** Jin-Feng Zhao, Natalia Shpiro, Gajanan Sathe, Abigail Brewer, Thomas J. Macartney, Nicola T. Wood, Florentina Negoita, Kei Sakamoto, Gopal P. Sapkota

**Affiliations:** 1 Medical Research Council (MRC) Protein Phosphorylation & Ubiquitylation Unit, School of Life Sciences, University of Dundee, Dundee DD1 5EH, UK; 2 Novo Nordisk Foundation Center for Basic Metabolic Research, University of Copenhagen, 2200 Copenhagen, Denmark

**Keywords:** Health sciences, Medicine, Medical specialty, Precision medicine

## Abstract

Reversible phosphorylation of the transcription factor EB (TFEB) coordinates cellular responses to metabolic and other stresses. During nutrient replete and stressor-free conditions, phosphorylated TFEB is primarily localized to the cytoplasm. Stressor-mediated reduction of TFEB phosphorylation promotes its nuclear translocation and context-dependent transcriptional activity. In this study, we explored targeted dephosphorylation of TFEB as an approach to activate TFEB in the absence of nutrient deprivation or other cellular stress. Through an induction of proximity between TFEB and several phosphatases using the AdPhosphatase system, we demonstrate targeted dephosphorylation of TFEB in cells. Furthermore, by developing a heterobifunctional molecule BDPIC (bromoTAG-dTAG proximity-inducing chimera), we demonstrate targeted dephosphorylation of TFEB-dTAG through induced proximity to bromoTAG-PPP2CA. Targeted dephosphorylation of TFEB-dTAG by bromoTAG-PPP2CA with BDPIC at the endogenous levels is sufficient to induce nuclear translocation and some transcriptional activity of TFEB.

## Highlights

• AdPhosphatase-mediated dephosphorylation of TFEB triggers its nuclear translocation
• A bifunctional molecule bromoTAG-dTAG proximity-inducing chimera, BDPIC, developed
• BDPIC selectively and efficiently recruits bromoTAG-PPP2CA to TFEB-dTAG in cells
• Targeted dephosphorylation of TFEB via PPP2CA alters its localization and activity

## Graphical abstract

**Figure:**
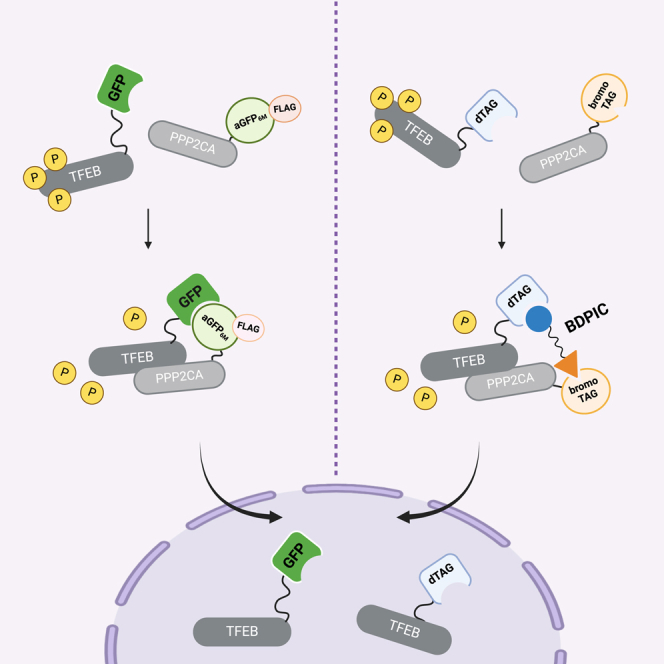

## Introduction

Transcription factor EB (TFEB) belongs to the microphthalmia/transcription factor E (MiT/TFE) family of basic helix-loop-helix leucine zipper (bHLH-Zip) transcription factors also comprising TFE3, MITF, and TFEB.^1^^,^^2^^,^^3^ Upon translocation to the nucleus, TFEB directly binds to a well-defined DNA binding element known as coordinated lysosomal expression and regulation (CLEAR) motif, which is conserved in the proximal promoters of numerous components of the lysosome and autophagy related genes.^1^^,^^2^^,^^3^ Emerging evidence suggests TFEB also plays a crucial role in the control of metabolic processes in response to nutrient and energy stress.^4^^,^^5^^,^^6^^,^^7^ Given the breadth of biological processes that TFEB controls, its dysregulation is linked to many human diseases, including cardiovascular diseases, metabolic syndromes, cancers, and neurodegeneration.^8^^,^^9^^,^^10^ The transcriptional activity of TFEB is tightly controlled and is regulated by post-translational modifications, primarily through reversible phosphorylation.^11^^,^^12^^,^^13^^,^^14^^,^^15^ Phosphorylation status of TFEB on multiple different residues, which is regulated via concerted actions of different kinases and phosphatases depending on specific environmental and signaling cues, is known to regulate its nuclear cytoplasmic shuttling, interaction with other partners and, ultimately, transcriptional activity.^11^^,^^12^^,^^13^^,^^14^ Under nutrient replete and stressor-free conditions, serine/threonine kinase mTORC1 is involved in the phosphorylation of TFEB at S122, S142 and S211 and promotes its retention in the cytoplasm.^16^^,^^17^^,^^18^^,^^19^^,^^20^ Phosphorylation of TFEB at S211 is known to promote 14-3-3 binding, leading to the retention of TFEB in the cytoplasm.^20^ Upon inactivation of mTORC1 under amino acid and growth factor starvation or pharmacological inhibition, TFEB phosphorylation is blocked, which leads to its dissociation from 14-3-3 and translocation from cytosol to nucleus, where it initiates its transcriptional activity.^20^ Activation of AMP-activated protein kinase (AMPK) also promotes TFEB dephosphorylation and nuclear translocation,^21^^,^^22^^,^^23^ which has recently been proposed to be mediated through direct phosphorylation of folliculin-interacting protein 1 (FNIP1) and subsequent suppression of the function of the folliculin (FLCN)–FNIP1 complex.^4^ Protein phosphatases PP2A and calcineurin (also known as PPP3 or PP2B) have been reported to dephosphorylate and activate TFEB upon oxidative stress and enhanced calcium signaling, respectively.^24^^,^^25^ Collectively, altering TFEB phosphorylation is central to its subcellular distribution and transcriptional activity.

Targeted dephosphorylation is an emerging modality that allows for a precise substrate-level control of intracellular proteins.^26^^,^^27^^,^^28^^,^^29^^,^^30^ The modality relies on redirecting protein phosphatases to specific proteins of interest (POI) through proximity induction. Parallels can be drawn from targeted protein degradation modality, where E3 ubiquitin ligases are redirected to neosubstrates for target protein degradation.^31^^,^^32^ Indeed, small molecule degraders, such as proteolysis targeting chimeras (PROTACs) and molecular glues, which facilitate proximity between E3 ubiquitin ligases and POIs inside cells to cause POI degradation, have entered clinical trials or are already in clinical use.^32^^,^^33^^,^^34^^,^^35^^,^^36^ Substrate level phospho-protein control can be elicited through inhibition of upstream kinases or activation of phosphatases. Significant research efforts have led to the development of many specific protein kinase inhibitors, some of which are in clinical use, and some phosphatase activators for use in research.^37^^,^^38^ However, as many kinases and phosphatases control multiple substrates, and the kinase inhibitors often have off-target effects,^39^^,^^40^^,^^41^^,^^42^ achieving a substrate-level phospho-control by using kinase inhibitors or phosphatase activators is extremely challenging. Targeted dephosphorylation of phospho-POI by recruiting a specific phosphatase can overcome these challenges. We have previously revealed that targeted dephosphorylation of phospho-POI can be achieved by utilizing the affinity-directed phosphatase (AdPhosphatase) system, which redirects phosphatase activity to a specific phospho-POI through high-affinity polypeptide POI binders.^30^ Protein phosphatase 1 (PP1)-recruiting peptide linked to inhibitors of protein kinase Akt was shown to cause reduced phosphorylation of Akt.^26^ Another study reported targeted dephosphorylation by developing heterobifunctional small molecules, such as PhosTAC7 (phosphorylation targeting chimera 7) that induces proximity between a dTAG (FKBP12^F36V^) and a Halo-Tag, to demonstrate that PhosTAC7 induced a reduction in phosphorylation of phospho-Halo-PDCD4, -Halo-FOXO3, or -Halo-tau through dTAG-PP2A A (protein phosphatase 2A A scaffolding subunit).^27^^,^^29^ Other studies have recently utilized similar bivalent molecule-based induced-proximity platforms for targeted loss in phosphorylation of phospho-POIs.^28^^,^^29^ Nonetheless, the above studies utilizing small bivalent molecules for targeted dephosphorylation have not definitively established reliance of the observed loss of POI phosphorylation on the catalytic activity of the recruited phosphatases.^27^^,^^28^^,^^29^

Given that multiple signaling events, such as inhibitors of mTOR or activators of AMPK pathways, converge on TFEB and promote its nuclear localization and transcriptional activity through a reduction in phospho-TFEB levels, targeted dephosphorylation of TFEB could in principle mimic the effects of these signaling events. Since TFEB activity stimulates autophagy and lysosomal activity, among other functions, if targeted dephosphorylation of TFEB leads to its activation, then it could offer a promising innovative strategy for therapeutic targeting against lysosomal-related diseases, such as neurodegenerative diseases, cystinosis, acute kidney injury, and diabetic nephropathy. Furthermore, such an approach would also allow one to investigate the functions of phosphorylation of TFEB on different residues. In this study, by employing various induced-proximity platforms, we set out to investigate the feasibility of targeted dephosphorylation of TFEB and its consequences on TFEB localization and transcriptional activity. With the AdPhosphatase technology, we show a robust targeted dephosphorylation of phospho-TFEB through recruitment of several protein phosphatases and demonstrate that this promotes the nuclear translocation of TFEB. Additionally, by using the heterobifunctional small molecule, the bromoTAG-dTAG proximity-inducing chimera (BDPIC), that we developed, we demonstrate that redirecting bromoTAG-PPP2CA (protein phosphatase 2 catalytic subunit A) to TFEB-dTAG leads to an inducible dephosphorylation of TFEB-dTAG, its nuclear translocation and transcription of TFEB target genes.

## Results

### AdPhosphatases aGFP_6M_-PPP1CA and aGFP_6M_-PPP2CA mediate the dephosphorylation of phospho-TFEB-GFP

**Figure 1:**
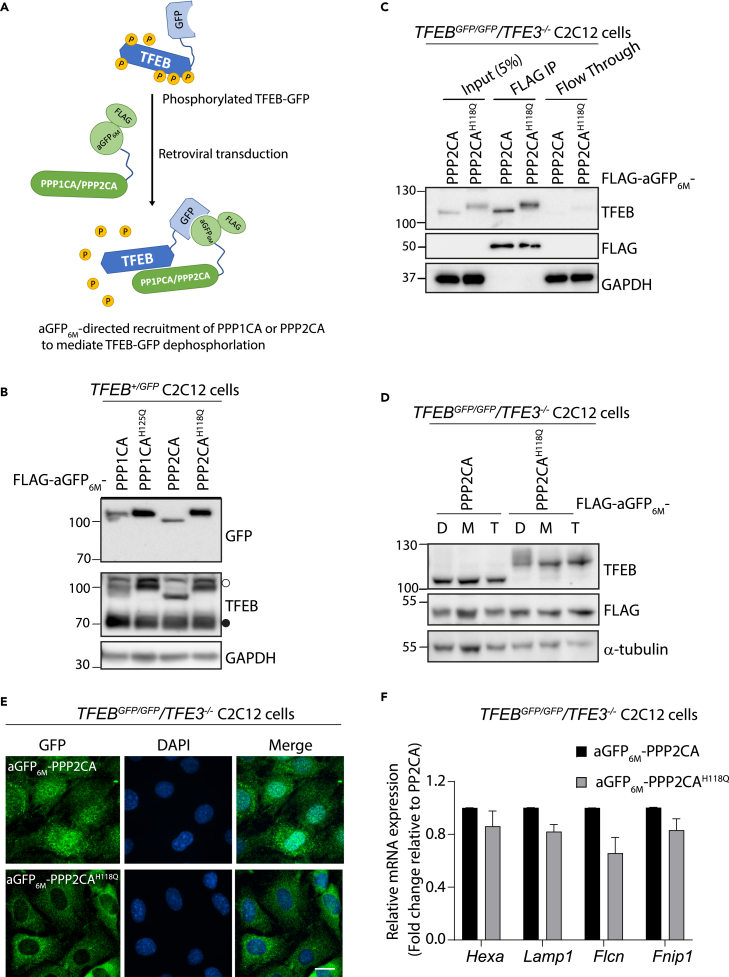
aGFP_6M_-AdPhosphatases dephosphorylate phospho-TFEB-GFP (A) Schematic representation of antigen-stabilized anti-GFP nanobody (aGFP_6M_)-directed recruitment of either PPP1CA or PPP2CA to C-terminal GFP-tagged TFEB (TFEB-GFP) to mediate TFEB-GFP dephosphorylation. (B) *TFEB*^*+/GFP*^ heterozygous C2C12 myoblasts stably expressing FLAG-aGFP_6M_-PPP1CA, catalytically inactive FLAG-aGFP_6M_-PPP1CA^H125Q^, FLAG-aGFP_6M_-PPP2CA or catalytically inactive FLAG-aGFP_6M_-PPP2CA^H118Q^ were lysed and subjected to immunoblot analysis. ○, TFEB-GFP; ●, unmodified endogenous TFEB. (C) *TFEB*^*GFP/GFP*^*/TFE3*^*−/−*^ C2C12 myoblasts stably expressing FLAG-aGFP_6M_-PPP2CA or FLAG-aGFP_6M_-PPP2CA^H118Q^ were lysed and lysates (1 mg protein) subjected to anti-FLAG immunoprecipitation (IP) prior to immunoblot analysis on input extracts (25 μg protein), IPs and post-IP flow-through extracts (25 μg protein) as indicated. (D) *TFEB*^*GFP/GFP*^*/TFE3*^*−/−*^ C2C12 myoblasts stably expressing FLAG-aGFP_6M_-PPP2CA or FLAG-aGFP_6M_-PPP2CA^H118Q^ were treated with vehicle (0.1% DMSO; D), MK-8722 [M] (10 μM) or Torin 1 [T] (100 nM) for 4 h prior to lysis and subjected to immunoblot analysis as indicated. (E) Immunofluorescence staining of GFP were performed in *TFEB*^*GFP/GFP*^*/TFE3*^*−/−*^ C2C12 myoblasts stably expressing FLAG-aGFP_6M_-PPP2CA or FLAG-aGFP_6M_-PPP2CA^H118Q^. Nucleus was stained with DAPI. Scale bars, 10 μm. Representative images included. (F) The expression of *Hexa*, *Lamp1*, *Flcn* and *Fnip1* transcripts was measured by RT-qPCR in *TFEB*^*GFP/GFP*^*/TFE3*^*−/−*^ C2C12 myoblasts stably expressing FLAG-aGFP_6M_-PPP2CA or Flag-aGFP_6M_-PPP2CA^H118Q^. All data shown in (B–F) are representative of 3 independent experiments. All quantitative data are mean ± SEM from 3 independent experiments. Statistical analysis involved t-test with Mann-Whitney test.

Under nutrient-replete conditions TFEB is phosphorylated by various kinases and resides in the cytoplasm, which results in a heterogeneous, smeared signal upon immunoblotting with an anti-TFEB antibody (Figures S1A and S1B). Upon its dephosphorylation by subjecting extracts to an *in vitro* dephosphorylation assay with λ-phosphatase, TFEB band exhibits a downward electrophoretic mobility shift of approximately 20kDa (Figures S1A and S1B). Therefore, the relative phosphorylation status of TFEB can be estimated by total TFEB immunoblots. Inhibition of mTORC1 (e.g., via nutrient starvation or small molecule inhibitors) or activation of AMPK (e.g., via energy stress such as hypoxia, or small molecules) results in a reduction in TFEB phosphorylation,^4^ which can be detected as a downward electrophoretic mobility shift (Figure S1B). This loss in phosphorylation of TFEB facilitates its nuclear translocation and transcriptional activation.^11^ We hypothesized that targeted dephosphorylation of phospho-TFEB could trigger its nuclear translocation and transcriptional activity, independently of nutrient/energy stress or small-molecule kinase modulators. We sought to test this in *TFEB*^*+/GFP*^ heterozygous cells generated using CRISPR/Cas9 genome editing, where introduction of the FLAG-aGFP_6M_-PPP1CA or -PPP2CA AdPhosphatases,^30^ would redirect the phosphatase activity to phospho-TFEB-GFP, but not untagged, wild-type (WT) phospho-TFEB, for targeted TFEB dephosphorylation (Figure 1A). Transduction of C2C12 *TFEB*^*+/GFP*^ cells with FLAG-aGFP_6M_-PPP1CA and FLAG-aGFP_6M_-PPP2CA retroviruses showed a downward electrophoretic mobility shift of TFEB-GFP, but not WT TFEB, compared with cells transduced with retroviruses encoding the catalytically inactive mutants of FLAG-aGFP_6M_-PPP1CA^H125Q^ and FLAG-aGFP_6M_-PPP2CA^H118Q^ AdPhosphatases (Figure 1B). These results suggest that the targeted nature of TFEB-GFP dephosphorylation is reliant on phosphatase activity of PP1CA or PP2CA and that the exogenous expression of PP1CA or PP2CA does not appear to impact the phosphorylation status of endogenous TFEB. Moreover, the downward electrophoretic mobility shift of TFEB-GFP observed in cells transduced with FLAG-aGFP_6M_-PPP2CA was more pronounced compared to those transduced with FLAG-aGFP_6M_-PPP1CA (Figure 1B) and that caused by incubation of extracts *in vitro* with λ-phosphatase (Figure S1A). The fact that human PPP1CA and PPP2CA AdPhosphatases cause dephosphorylation of TFEB in C2C12 cells derived from mice implies the conserved nature of PP1 and PP2A holoenzyme complexes between human and mouse. To probe targeted dephosphorylation of TFEB-GFP at the endogenous level further, we generated C2C12 *TFEB*^*GFP/GFP*^*/TFE3*^*−/−*^ cells, where homozygous TFEB-GFP knock-in was followed up with TFE3 knockout using CRISPR/Cas9 genome editing (Figure S2A), to avoid any potential compensatory function by TFE3. In these cells, we observed a robust targeted dephosphorylation of TFEB-GFP by FLAG-aGFP_6M_-PPP2CA but not FLAG-aGFP_6M_-PPP2CA^H118Q^ (Figure 1C) and further showed that TFEB-GFP co-precipitated with FLAG-aGFP_6M_-PPP2CA runs at a substantially lower molecular weight compared to that co-precipitated with FLAG-aGFP_6M_-PPP2CA^H118Q^ (Figure 1C), demonstrating the targeted and phosphatase-activity dependent nature of dephosphorylation. We also examined whether other AdPhosphatases, including PP2B (PPP3CA, PPP3CB, and PPP3CC) and PP5 (PPP5C), have a similar effect on TFEB-GFP dephosphorylation (Figures S3A–S3C). Targeted recruitment of various aGFP_6M_-PP2B isoforms and -PP5 also promoted TFEB-GFP dephosphorylation to varying extents, whereas the corresponding catalytically inactive mutants did not (Figures S3A–S3C).

Next, we compared the change in the electrophoretic mobility shift in TFEB-GFP caused by AdPhosphatase-mediated dephosphorylation against changes caused by pharmacological activation of AMPK^21^^,^^22^^,^^23^ or inhibition of mTORC1.^16^^,^^17^^,^^18^^,^^19^^,^^20^ When C2C12 *TFEB*^*GFP/GFP*^*/TFE3*^*−/−*^ cells transduced with catalytically inactive FLAG-aGFP_6M_-PPP2CA^H118Q^ AdPhosphatase retroviruses were treated with an allosteric AMPK activator (MK-8722) or mTORC1 inhibitor (Torin 1) for 4 h, a downward electrophoretic mobility shift in TFEB-GFP was apparent compared to vehicle (DMSO) control (Figure 1D). However, FLAG-aGFP_6M_-PPP2CA AdPhosphatase caused a larger electrophoretic mobility shift of TFEB-GFP regardless of treatment (Figure 1D), suggesting additional dephosphorylation of phospho-TFEB residues beyond those regulated by AMPK activation or mTORC1 inhibition. Analysis of TFEB-GFP in anti-FLAG IPs from *TFEB*^*GFP/GFP*^*/TFE3*^*−/−*^ cells expressing FLAG-aGFP_6M_-PPP2CA AdPhosphatase compared to those expressing FLAG-aGFP_6M_-PPP2CA^H118Q^ by mass spectrometry revealed the absence of multiple phospho-residues, including S113 (S114 in human), T132 (T133 in human) and/or S141 (S142 in human), T329, T330 or S331 (T330, T331 or S332 in human), S466 and/or S468 (S467 and/or S469 in human) (Figure S4), suggesting that these sites on TFEB were dephosphorylated by the AdPhosphatase. However, many other reported phospho-residues on TFEB,^15^ such as S210 (S211 in human), were not detected in our proteomic experiments suggesting incomplete coverage. Fluorescence microscopy analysis of *TFEB*^*GFP/GFP*^*/TFE3*^*−/−*^ cells expressing FLAG-aGFP_6M_-PPP2CA displayed a predominantly nuclear TFEB-GFP signal merging with DAPI, while cells expressing FLAG-aGFP_6M_-PPP2CA^H118Q^ showed cytoplasmic TFEB-GFP localization (Figure 1E). Finally, we assessed the transcription of reported TFEB target genes in *TFEB*^*GFP/GFP*^*/TFE3*^*−/−*^ cells upon AdPhosphatase-mediated dephosphorylation and nuclear translocation of TFEB-GFP. Despite the robust nuclear translocation of TFEB-GFP caused by FLAG-aGFP_6M_-PPP2CA (Figure 1E), we did not observe significant increase in transcription of known TFEB-regulated genes, including *Hexa, Lamp1, Flcn*, *and Fnip1*,^21^^,^^43^ compared to cells expressing FLAG-aGFP_6M_-PPP2CA^H118Q^, where TFEB-GFP remained in the cytosol (Figure 1F). We also tested whether dephosphorylation of TFEB by other AdPhosphatases that cause varying levels of dephosphorylation leads to changes in TFEB-dependent transcriptional activity (Figures S3A–S3C). Like the FLAG-aGFP_6M_-PPP2CA AdPhosphatase, we did not detect any significant changes in transcription of TFEB-regulated genes with FLAG-aGFP_6M_-PPP3C isoforms or -PPP5C AdPhosphatases (Figures S3D–S3F). To rule out the possibility that the constitutive expression of FLAG-aGFP_6M_-PPP2CA in C2C12 *TFEB*^*GFP/GFP*^*/TFE3*^*−/−*^ cells might have dampened TFEB-mediated gene transcription by activating feedback pathways, we developed a tetracycline-inducible AdPhosphatase expression system for inducible dephosphorylation of TFEB-GFP (Figure S5). Treatment of these cells with doxycycline-induced increased expressions of FLAG-aGFP_6M_-PPP2CA or FLAG-aGFP_6M_-PPP2CA^H118Q^ in a time- and dose-dependent manner (Figure S5A). Concurrently, a time- and dose-dependent TFEB-GFP dephosphorylation, as seen by a downward electrophoretic mobility shift, was evident in cells treated with doxycycline to induce the expression of FLAG-aGFP_6M_-PPP2CA, but not FLAG-aGFP_6M_-PPP2CA^H118Q^ (Figure S5A). The TFEB-GFP dephosphorylation mobility shift caused by doxycycline treatment to induce the FLAG-aGFP_6M_-PPP2CA AdPhosphatase expression was bigger than that caused by MK-8722 or Torin 1 treatment compared to DMSO treated controls in the absence of doxycycline or cells treated with DMSO and doxycycline to induce FLAG-aGFP_6M_-PPP2CA^H118Q^ (Figure S5B). Even under these conditions, there was no significant difference in the expression of *Hexa, Flcn*, *and Fnip1* transcripts between DMSO and doxycycline treatments in either FLAG-aGFP_6M_-PPP2CA or FLAG-aGFP_6M_-PPP2CA^H118Q^ cells (Figure S5C). This could be explained by multiple possibilities: i. since TFEB-dependent transcription is highly cell-context dependent, the clonal C2C12 cells we used do not respond to activation of TFEB; ii. targeted dephosphorylation of TFEB-GFP and its nuclear translocation alone is not sufficient for driving the transcription of TFEB target genes; iii. the constitutive interaction between FLAG-aGFP_6M_-PPP2CA and TFEB-GFP potentially interferes with the assembly of transcriptional complexes with other cofactors at the CLEAR motif on the target gene promoter for the initiation of transcriptional activity. Although each of these possibilities could be elaborated further, to circumvent the constitutive nature of the AdPhosphatase system, we instead focused on developing a chemically inducible system for targeted dephosphorylation by utilizing proximity-induction through small bivalent molecules.

### Development of small heterobifunctional molecule BDPIC to induce proximity between FKBP12^F36V^ (dTAG) and Brd4BD2^L387A^ (bromoTAG)

**Figure 2:**
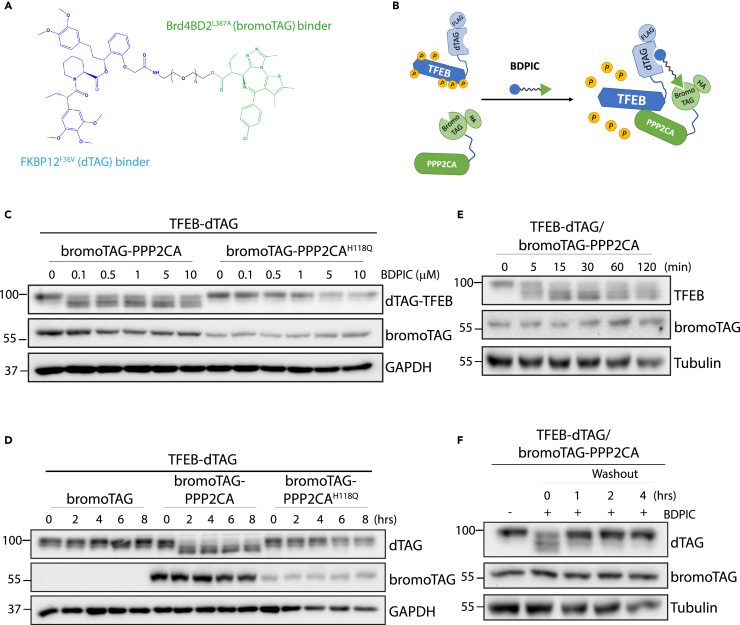
Targeting TFEB-dTAG dephosphorylation through bromoTAG-dTAG proximity-inducing chimera (BDPIC)-mediated recruitment of bromoTAG-PPP2CA (A) Structure of BDPIC with FKBP12^F36V^ (dTAG) and Brd4BD2^L387A^ (bromoTAG) binding warheads connected with the indicated PEG linker. (B) Schematic representation of BDPIC-mediated induction of proximity between TFEB-dATG-FLAG and HA-bromoTAG-PPP2CA. (C–F) U2OS cells stably co-expressing TFEB-dTAG-FLAG and HA-bromoTAG-empty, HA-bromoTAG-PPP2CA or HA-bromoTAG-PPP2CA^H118Q^ were generated. (C) Cells were treated with BDPIC at indicated concentrations for 24 h before lysis and extracts (10 μg protein) were subjected to immunoblot analysis as indicated. (D and E) Cells were treated with 100 nM BDPIC for indicated times prior to lysis. TFEB and PPP2CA protein abundance was analyzed by immunoblotting using the indicated antibodies. (F) Cells were treated with 100 nM BDPIC for 2 h. BDPIC was washed off by PBS and fresh culture media without BDPIC was added to cells. Cells were subsequently cultured for indicated times prior to lysis and immunoblot analysis was performed as indicated. All data (C–F) are representative of 3 independent experiments.

We developed a heterobifunctional small molecule with the established warheads binding to dTAG on one end^44^ and bromoTAG on the other end^45^ connected through 3-polyethylene glycol (PEG) linker (Figure 2A). We named this molecule BDPIC (bromoTAG-dTAG proximity-inducing chimera). We reasoned that in cells expressing bromoTAG-PPP2CA and TFEB-dTAG, this molecule would induce the proximity between them potentially allowing PPP2CA to dephosphorylate TFEB (Figure 2B). We employed the robust downward electrophoretic mobility shift of TFEB as a reporter for dephosphorylation. We generated U2OS cells stably expressing TFEB-dTAG-FLAG and HA-bromoTAG-PPP2CA or -PPP2CA^H118Q^ by means of retroviral transduction. When cells were treated with increasing concentrations of BDPIC for 24 h, a robust downward electrophoretic shift of TFEB-dTAG-FLAG was evident in cells co-expressing HA-bromoTAG-PPP2CA at all doses (100–10000 nM) of BDPIC compared to DMSO treated control (Figure 2C). Strikingly, no apparent mobility shift was detected in cells co-expressing HA-bromoTAG-PPP2CA^H118Q^ (Figure 2C), suggesting that the phosphatase activity of PPP2CA was essential for BDPIC-induced mobility shift of TFEB-dTAG-FLAG. A 2–8 h time-course treatment of cells with 100 nM BDPIC to assess the kinetics of dephosphorylation showed complete dephosphorylation of TFEB-dTAG-FLAG at 2 h, which was sustained until 8 h, in cells co-expressing HA-bromoTAG-PPP2CA (Figure 2D). No mobility shift of TFEB-dTAG-FLAG was observed with BDPIC treatment at any timepoints in cells co-expressing HA-bromoTAG alone or HA-bromoTAG-PPP2CA^H118Q^ (Figure 2D). A shorter time-course experiment revealed that BDPIC-mediated dephosphorylation of TFEB-dTAG-FLAG in cells co-expressing HA-bromoTAG-PPP2CA started as early as 5 min and peaked at around 15–30 min (Figure 2E). Next, we explored if BDPIC-mediated dephosphorylation of TFEB-dTAG-FLAG is reversible by undertaking a washout experiment. Cells co-expressing TFEB-dTAG-FLAG and HA-bromoTAG-PPP2CA were treated with 100 nM BDPIC for 2 h and washed twice with PBS and replaced with fresh culture medium without BDPIC. The TFEB-dTAG-FLAG phospho-dependent electrophoretic mobility shift recovered almost completely back to DMSO-treated conditions within 1 h of BDPIC washout and completely after 4 h following BDPIC washout (Figure 2F). As we observed that the level of PPP2CA^H118Q^ mutant was much lower than WT PPP2CA (Figures 2C and 2D), to rule out the possibility that it was the higher level of WT PPP2CA rather than catalytic activity that caused TFEB dephosphorylation in response to BDPIC, an extensive retroviral transduction titration was carried out to normalize the protein levels of HA-bromoTAG-PPP2CA and HA-bromoTAG-PPP2CA^H118Q^ mutant (Figure S6). Under conditions where levels were normalized, BDPIC-induced dephosphorylation of TFEB-dTAG is specifically observed in cells expressing HA-bromoTAG-PPP2CA, but not those expressing HA-bromoTAG-PPP2CA^H118Q^ (Figure S6). To demonstrate that the nature of the tag on the PPP2CA or TFEB did not influence the efficacy of BDPIC-mediated dephosphorylation, we switched tags by introducing bromoTAG on TFEB and dTAG on PPP2CA or PPP2CA^H118Q^ (Figure S7A). Indeed, BDPIC induced TFEB-bromoTAG dephosphorylation in cells expressing dTAG-PPP2CA but not dTAG or dTAG- PPP2CA^H118Q^ controls (Figure S7B). Collectively, these data validate BDPIC’s cell permeability, ability to engage both dTAG and bromoTAG and induce their proximity, and the reversible nature of the BDPIC-induced proximity.

We also developed another bivalent small molecule with the established warheads binding to dTAG on one end and Halo-tag on the other end connected with 4-PEG linker, named HDPIC (Halo-tag-dTAG proximity-inducing chimera) (Figures S8A and S8B). Increasing doses of HDPIC treatment failed to cause TFEB-Halo electrophoretic mobility shift in U2OS cells co-expressing dTAG-PPP2CA compared to DMSO control, and also did not cause any shift in control cells or those co-expressing dTAG-PPP2CA^H118Q^ (Figure S8C). Interestingly, HDPIC had a destabilizing effect on TFEB-Halo in all conditions with increasing concentrations of HDPIC leading to lower levels of TFEB-Halo (Figure S8C). Recently, PhosTAC7, a compound that resembles HDPIC but is structurally distinct, was reported to recruit dTAG-PP2A-A subunit to different Halo-tagged proteins, including PDCD4 and FOXO3a, for proximity-induced dephosphorylation.^27^ When we employed increasing amounts of PhosTAC7 in U2OS cells co-expressing TFEB-Halo and dTAG-PPP2CA or dTAG-PPP2CA^H118Q^, we did not observe any electrophoretic mobility shift of TFEB-Halo under any conditions. Like HDPIC, increasing concentrations of PhosTAC7 also caused a reduction in levels of TFEB-Halo (Figure S8D). Since neither HDPIC nor PhosTAC7 appeared to cause any TFEB-Halo dephosphorylation by dTAG-PPP2CA, and instead seemed to cause it to be destabilized, we decided not to use these molecules for further studies.

### BDPIC-mediated dephosphorylation of endogenous TFEB-dTAG through induced-proximity with bromoTAG-PPP2CA

**Figure 3:**
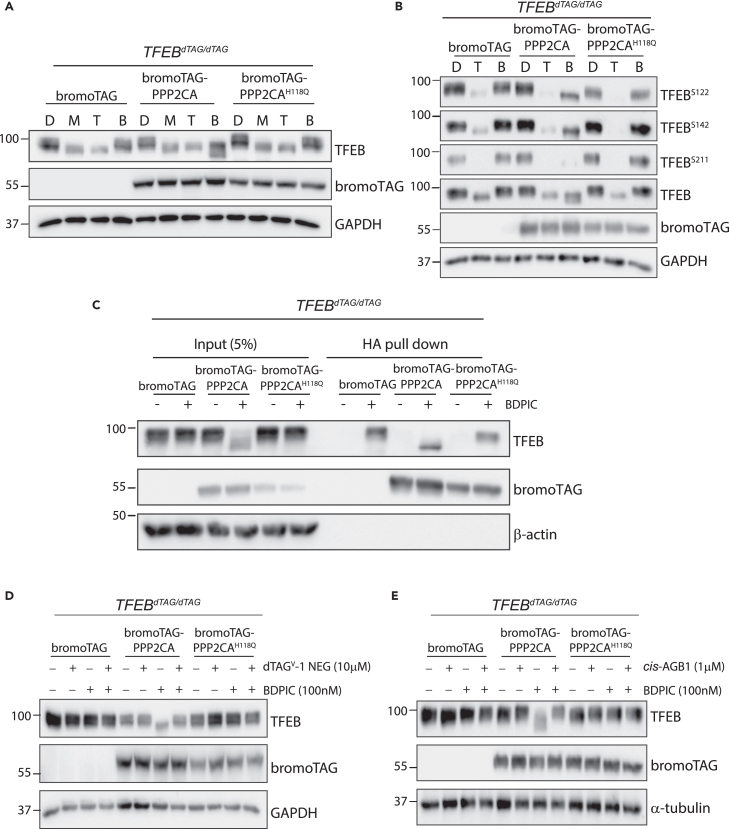
BDPIC-mediated targeted dephosphorylation of endogenous TFEB-dTAG in *TFEB*^*dTAG/dTAG*^ knock-in U2OS cells through overexpressed bromoTAG-PPP2CA (A–E) *TFEB*^*dTAG/dTAG*^ U2OS cells stably expressing HA-bromoTAG-empty, HA-bromoTAG-PPP2CA or HA-bromoTAG-PPP2CA^H118Q^ were generated. (A) Cells were treated with DMSO [D], MK-8722 [M] (10 μM), Torin 1 [T] (100 nM) or BDPIC [B] (100 nM) for 2 h before lysis and extracts (25 μg protein) were subjected to immunoblot analysis as indicated. (B) Cells were treated with DMSO [D], 100 nM Torin 1 [T] or BDPIC [B] for 2 h prior to lysis. Extracts (25 μg protein) were subjected to immunoblot analysis as indicated. (C) Cells were treated with 100 nM BDPIC for 2 h. DSP-crosslinking was performed prior to lysis. Cell extracts were subjected to anti-HA pull down. TFEB-dTAG and HA-bromoTAG-PPP2CA proteins were analyzed by immunoblotting. Cells were treated with excess amount (10 μM) of dTAG^V^-1-NEG PROTAC compound (D) or (1 μM) *cis*-AGB1 (E) for 30 min prior to treating cells with 100 nM BDPIC. After 2 h, cells were lysed and subjected to immunoblot with the indicated antibodies. All data (A–E) are representative of 3 independent experiments.

To investigate the consequences of targeted dephosphorylation of TFEB, we generated *TFEB*^*dTAG/dTAG*^ homozygous knock-in U2OS cells by CRISPR/Cas9 genome editing (Figure S2B). Treatment of *TFEB*^*dTAG/dTAG*^ U2OS cells stably expressing HA-bromoTAG alone, HA-bromoTAG-PPP2CA or HA-bromoTAG-PPP2CA^H118Q^ with DMSO did not affect TFEB-dTAG mobility. In contrast, treatment of the *TFEB*^*dTAG/dTAG*^ U2OS cells with MK-8722 (to activate AMPK) or Torin 1 (to inhibit mTORC1) caused the expected downward mobility shift of TFEB-dTAG (Figure 3A). This indicates that incorporation of dTAG on endogenous TFEB or overexpressing HA-bromoTAG, HA-bromoTAG-PPP2CA or HA-bromoTAG-PPP2CA^H118Q^ does not appear to affect homeostatic levels of TFEB phosphorylation (Figure 3A). Treatment of *TFEB*^*dTAG/dTAG*^ U2OS cells stably expressing HA-bromoTAG-PPP2CA, but not those expressing HA-bromoTAG and HA-bromoTAG-PPP2CA^H118Q^, with BDPIC caused dephosphorylation of TFEB-dATG, to a greater extent than that caused by MK-8722 or Torin 1 treatment (Figure 3A). There was a marked decrease in phosphorylation of TFEB at Ser122, Ser142 and Ser211 in cells stably expressing HA-bromoTAG-PPP2CA compared to those expressing HA-bromoTAG and HA-bromoTAG-PPP2CA^H118Q^ following treatment with BDPIC (Figure 3B). The fact that BDPIC mediates targeted dephosphorylation of TFEB-dTAG by HA-bromoTAG-PPP2CA implies a ternary complex formation between TFEB-dTAG and HA-bromoTAG-PPP2CA in the presence of BDPIC. To examine this, *TFEB*^*dTAG/dTAG*^ U2OS cells stably expressing HA-bromoTAG alone, HA-bromoTAG-PPP2CA or HA-bromoTAG-PPP2CA^H118Q^ and treated with either DMSO or BDPIC were subjected to anti-HA immunoprecipitation (IP) (Figure 3C). TFEB-dATG was co-precipitated in IPs of HA-bromoTAG, HA-bromoTAG-PPP2CA and HA-bromoTAG-PPP2CA^H118Q^ when cells were treated with BDPIC but not DMSO (Figure 3C). Moreover, the faster-migrating form of TFEB-dTAG was only detected in the anti-HA IPs from cells expressing HA-bromoTAG-PPP2CA treated with BDPIC (Figure 3C). We also performed competition assays using excess amounts of dTAG^V^-1-NEG (inactive enantiomer of the dTAG-V1 PROTAC that shares the same dTAG binder as BDPIC but is unable to recruit VHL for dTAG degradation),^46^ or *cis*-AGB1 (inactive enantiomer of AGB1 PROTAC that shares the same bromoTAG binder as BDPIC but is unable to recruit VHL to degrade bromoTAG protein).^45^ Pre-treatment of cells with excess of dTAG^V^-1-NEG or *cis*-AGB1 compounds prevented the dephosphorylation of TFEB-dATG in *TFEB*^*dTAG/dTAG*^ U2OS cells stably expressing HA-bromoTAG-PPP2CA caused by BDPIC (Figures 3D and 3E). This data confirms that BDPIC-mediated targeted dephosphorylation of TFEB-dATG relies on the binding of BDPIC with TFEB-dTAG.

**Figure 4:**
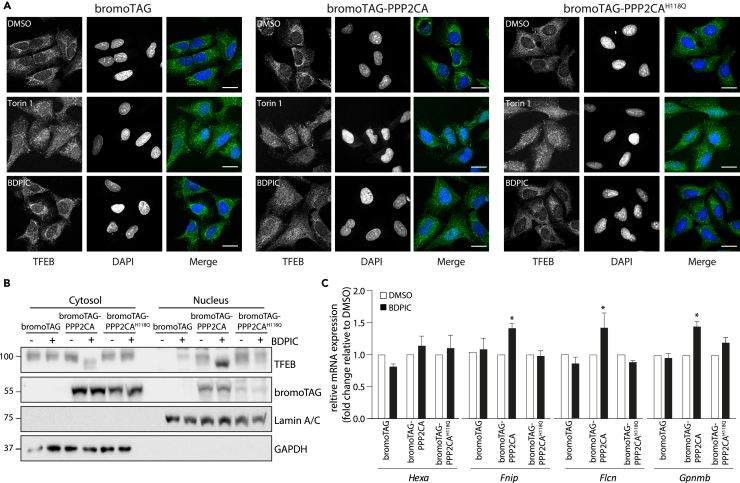
BDPIC-mediated targeted dephosphorylation of TFEB-dTAG promotes its nuclear translocation and transcriptional activation of some reported target genes (A) Immunostaining of TFEB was performed in *TFEB*^*dTAG/dTAG*^ knock-in U2OS cells stably expressing HA-bromoTAG, HA-bromoTAG-PPP2CA or HA-bromoTAG-PPP2CA^H118Q^ treated with DMSO, Torin 1 (100 nM) or BDPIC (100 nM) for 2 h prior to fixation. Nucleus was stained with DAPI. Scale bars, 10 μm. Data are representative of 3 independent experiments. (B) *TFEB*^*dTAG/dTAG*^ knock-in U2OS cells stably expressing HA-bromoTAG, HA-bromoTAG-PPP2CA or HA-bromoTAG-PPP2CA^H118Q^ were treated with DMSO or 100 nM BDPIC for 2 h prior to lysis. Cytoplasmic and nuclear fractions were isolated and subjected to immunoblot analysis with the indicated antibodies. Data are representative of 3 independent experiments. (C) *TFEB*^*dTAG/dTAG*^ knock-in U2OS cells were treated with DMSO or 100 nM BDPIC for 8 h prior to lysis. The expression of *Hexa*, *Fnip1*, *Flcn*, and *Gpnmb* transcripts was examined by RT-qPCR. All quantitative data are mean ± SEM from 3 independent experiments. ∗*p* < 0.05 compared with DMSO treatment. Statistical analysis involved t-test with Mann-Whitney test.

Next, we investigated whether BDPIC-mediated TFEB-dTAG dephosphorylation in *TFEB*^*dTAG/dTAG*^ knock-in U2OS cells can trigger its nuclear translocation and transcriptional activity. In *TFEB*^*dTAG/dTAG*^ U2OS cells stably expressing HA-bromoTAG-PPP2CA, but not in those expressing HA-bromoTAG or HA-bromoTAG-PPP2CA^H118Q^, BDPIC treatment caused a robust nuclear TFEB-dTAG immunostaining signal, which overlapped with DAPI staining, while DMSO treatment showed a predominantly cytoplasmic TFEB-dTAG staining in all cell lines (Figure 4A). Employed as a positive control, Torin 1 caused nuclear staining of TFEB-dTAG in all cell lines (Figure 4A). We also observed a larger amount of faster migrating TFEB-dTAG signal in the nuclear fractions of BDPIC-treated cells stably expressing HA-bromoTAG-PPP2CA than in the cytoplasmic fraction, while DMSO treatment did not cause increased nuclear accumulation of TFEB-dTAG nor did it induce TFEB-dTAG dephosphorylation (Figure 4B). In *TFEB*^*dTAG/dTAG*^ U2OS cells stably expressing HA-bromoTAG or HA-bromoTAG-PPP2CA^H118Q^ controls, neither DMSO nor BDPIC caused dephosphorylation or nuclear translocation of TFEB-dTAG (Figure 4B). We analyzed the mRNA expression of some known TFEB-target genes in *TFEB*^*dTAG/dTAG*^ U2OS cells stably expressing HA-bromoTAG, HA-bromoTAG-PPP2CA or HA-bromoTAG-PPP2CA^H118Q^ by RT-qPCR following 8 h of BDPIC treatment. We observed that compared to DMSO control, BDPIC treatment induced only a slight but significant increase in expression of *Fnip1, Flcn*,^21^^,^^43^ *and Gpnmb*^47^^,^^48^^,^^49^ transcripts in cells expressing HA-bromoTAG-PPP2CA but not in those expressing HA-bromoTAG or HA-bromoTAG-PPP2CA^H118Q^ (Figure 4C), suggesting that targeted dephosphorylation of TFEB-dTAG by BDPIC is potentially sufficient to cause transcriptional activation of TFEB. The transcription of *Hexa*, another TFEB-target gene, was not significantly affected by targeted dephosphorylation of TFEB-dTAG by BDPIC (Figure 4C). We also explored the impact of targeted dephosphorylation of TFEB-dTAG by BDPIC caused by the recruitment of various other phosphatases, including HA-bromoTAG-PPP3C isoforms and HA-bromoTAG-PP5C. Recruitment of HA-bromoTAG-PPP3CA, -PPP3CB or -PPP5C to TFEB-dTAG by BDPIC led to dephosphorylation of TFEB-dTAG to varying degrees, while the corresponding catalytically inactive mutants showed no such effects (Figures S9A and S9B). Moreover, induction of some of the reported TFEB target genes was modestly but significantly increased upon BDPIC-mediated dephosphorylation of TFEB-dTAG caused by HA-bromoTAG-PPP3CA, and -PPP3CB but not -PPP5C, while the catalytically inactive mutants did not cause any changes in TFEB-dTAG phosphorylation or induction of target genes (Figures S9A–S9E). The full scope of TFEB-mediated transcription of target genes in U2OS cells is currently not known. So, future work needs to establish whether BDPIC induces binding of TFEB-dTAG to the CLEAR motifs within the promoter region of *Fnip1, Flcn*, *and Gpnmb* genes in *TFEB*^*dTAG/dTAG*^ knock-in U2OS cells stably expressing HA-bromoTAG-PPP2CA to confirm that the modest increase in transcription we observe is indeed via TFEB-dTAG.

### BDPIC-mediated dephosphorylation of phospho-TFEB-dTAG through the recruitment of bromoTAG-PPP2CA at the endogenous level

**Figure 5:**
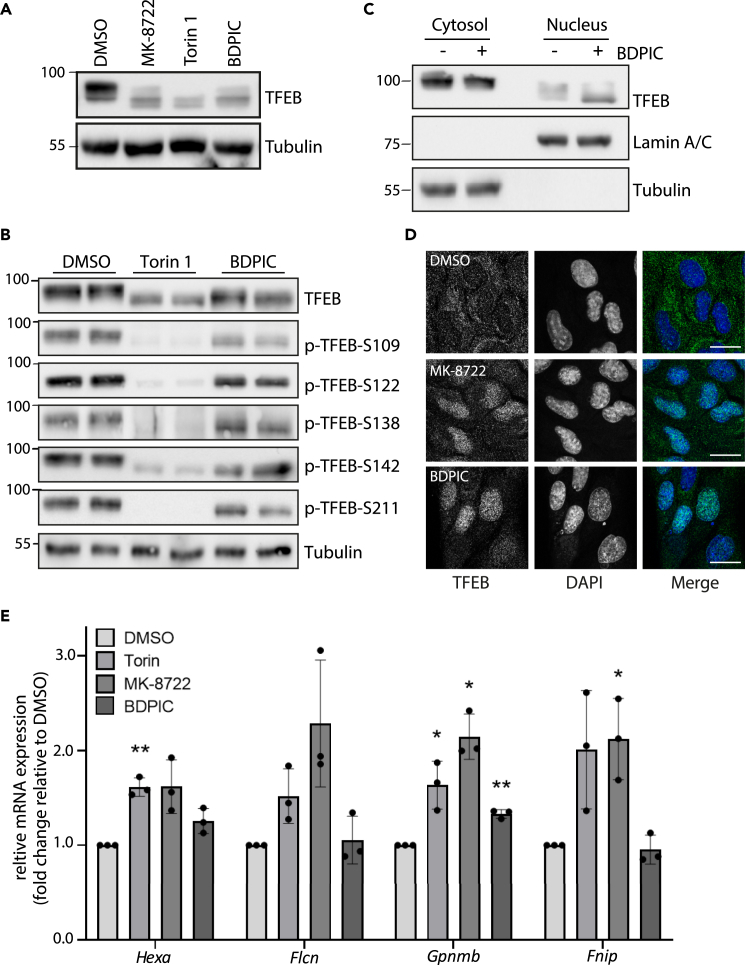
BDPIC-mediated targeted dephosphorylation of TFEB-dTAG via bromoTAG-PPP2CA at the endogenous level (A–E) *TFEB*^*dTAG/dTAG*^*/*^*bromoTAG/+*^*PPP2CA* U2OS cells were generated by CRISPR/Cas9 technology. (A) Cells treated with DMSO, MK-8722 (10 μM), Torin 1 (100 nM) or BDPIC (100 nM) for 2 h prior to lysis and extracts (25 μg protein) subjected to immunoblot analysis. (B) Cells were treated with either DMSO, 100 nM Torin 1 or 100 nM BDPIC for 2 h prior to lysis. Extracts (25 μg protein) were subjected to immunoblot analysis as indicated. (C) Cytoplasmic and nuclear fractions from cells treated with DMSO or 100 nM BDPIC for 2 h were extracted and subjected to immunoblot with the indicated antibodies. (D) Cells were treated with DMSO, MK-8722 (10 μM) or BDPIC (100 nM) for 2 h before fixation and immunostaining for TFEB. Nucleus was stained with DAPI. Scale bars, 10 μm. (A–D) All data representative of 3 independent experiments. (E) Cells were treated with DMSO, Torin 1 (100 nM), MK-8722 (10 μM) or BDPIC (100 nM) for 4 h prior to lysis. The expression of *Hexa*, *Flcn*, *Gpnmb*, and *Fnip1* transcripts was examined by RT-qPCR. All quantitative data are mean ± SD from 3 independent experiments, with statistical analysis involving paired t-tests to compare to DMSO treatment. ∗*p < 0.05* and ∗∗*p < 0.01* compared with DMSO treatment.

**Figure 6:**
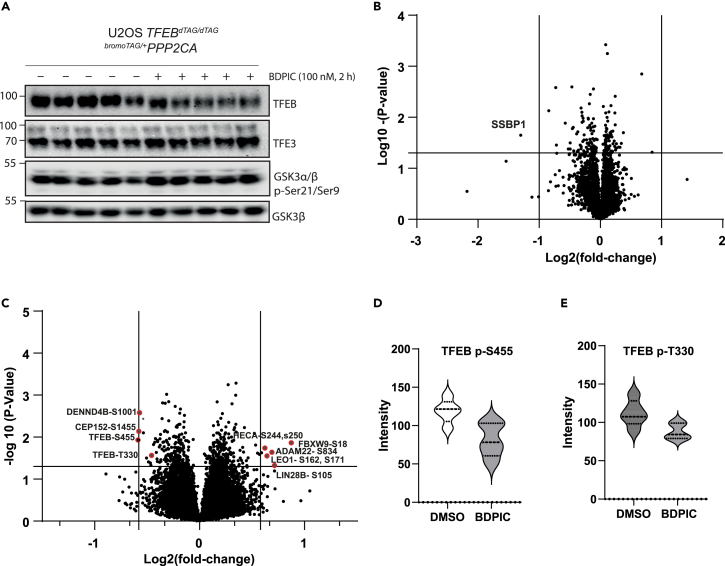
Global quantitative proteomic and phospho-proteomic analyses of BDPIC-mediated targeted dephosphorylation of TFEB-dTAG (A) *TFEB*^*dTAG/dTAG*^*/*^*bromoTAG/+*^*PPP2CA* U2OS cells were treated with either DMSO or 100 nM BDPIC for 2 h prior to lysis. Extracts (25 μg protein) were subjected to immunoblot analysis as indicated. (B) Volcano plot showing quantitative changes in the identified proteins. Data plotted represents log_2_ of the fold change in protein abundance in BDPIC-treated extracts normalized to DMSO-treated controls against log_10_ of the *p* value for each identified protein. The only protein showing a significant change of >2-fold is indicated. (C) Volcano plot showing global phospho-proteome alterations in BDPIC-treated cells compared to DMSO-treated controls. Data plotted represents log_2_ of the fold change of phospho-peptides identified in BDPIC-treated cell extracts normalized to DMSO-treated controls against log_10_ of the *p* value for each phospho-peptide. Significant alterations of >1.5-fold are indicated. (D and E) Violin plots of individual TFEB phospho-peptides identified by quantitative phospho-proteomic analysis.

We have demonstrated that induced proximity between endogenously knocked-in TFEB-dTAG and over-expressed HA-bromoTAG-PPP2CA causes dephosphorylation and transcriptional activation of TFEB on some target genes. However, to avoid the possibility of any unanticipated artifacts resulting from PPP2CA overexpression, such as dephosphorylation of native PPP2CA substrates and their physiological consequences, we generated *TFEB*^*dTAG/dTAG*^*/*^*bromoTAG/+*^*PPP2CA* double knock-in U2OS cells by employing CRISPR/Cas9 gene editing technology (Figures S2C–S2G). Treatment of these cells with 100 nM BDPIC resulted in a downward electrophoretic mobility shift of TFEB-dTAG, to a similar extent to that caused by the treatment of cells with MK-8722 or Torin 1, compared to DMSO treatment (Figure 5A), indicating that through BDPIC, endogenous bromoTAG-PPP2CA activity can be redirected to dephosphorylate TFEB-dTAG. However, this BDPIC-induced mobility shift of TFEB-dTAG at the endogenous level was smaller than that observed when bromoTAG-PPP2CA was overexpressed (Figure 3), potentially due to the low levels of bromoTAG-PPP2CA in *TFEB*^*dTAG/dTAG*^*/*^*bromoTAG/+*^*PPP2CA* cells, as we were only able to obtain heterozygous insertion of bromoTAG on a single PPP2CA allele (Figures S2C–S2G). BDPIC treatment of *TFEB*^*dTAG/dTAG*^*/*^*bromoTAG/+*^*PPP2CA* cells did not cause a mobility shift of the related unmodified transcription factor TFE3 at the endogenous level (Figures 6A and S10A). Similarly, redirecting endogenous bromoTAG-PPP2CA to dephosphorylate TFEB-dTAG with BDPIC did not appear to substantially alter the phosphorylation of GSK3 α/β at Ser21/9, which are endogenous targets of PPP2CA^50^ (Figure 6A). By immunoblotting, upon BDPIC treatment, we observed a slight reduction in the phosphorylation of TFEB-dTAG at Ser109, Ser122, Ser142 and Ser211, while there was no obvious change in the phosphorylation of Ser138 (Figure 5B). We also observed that upon BDPIC treatment, all of the faster migrating (dephosphorylated) pool of TFEB-dTAG was detected in the nuclear fraction, while the slower migrating (i.e., phosphorylated) pool resembling TFEB-dTAG from DMSO treated control cells remained in the cytosol (Figure 5C). By immunostaining, we were able to show that both MK-8722 and BDPIC induced a similar, robust nuclear localization of TFEB-dTAG, while cells treated with DMSO showed TFEB-dTAG staining predominantly in the cytosol (Figure 5D). We tested the transcription of TFEB target genes *Hexa*, *Flcn*, *Gpnmb*, *and Fnip1* in *TFEB*^*dTAG/dTAG*^*/*^*bromoTAG/+*^*PPP2CA* cells upon Torin 1, MK-8722 or BDPIC treatment (Figure 5E). Both Torin 1 and MK-8722 induced an increase in expression of all 4 genes by between 1.5- and 2-fold over DMSO-treated controls (Figure 5E). Under these conditions, BDPIC induced a 1.2-fold increase in transcription of *Gpnmb* over DMSO treated control but did not significantly alter the transcription of other genes (Figure 5E).

We tested whether BDPIC caused any cytotoxicity, especially at doses that were used to induce targeted dephosphorylation of TFEB-dTAG. Treatment of *TFEB*^*dTAG/dTAG*^*/*^*bromoTAG/+*^*PPP2CA* U2OS cells with BDPIC at concentrations ranging from 10 nM to 10 μM for 48 h did not show any effect on cell viability, even at the highest concentration of 10 μM (Figure S10D). Employed as a positive control, treatment of cells with MG132 for 48 h led to profound inhibition of cell proliferation as well as cell death (Figure S10D).

To determine the specificity of BDPIC-mediated TFEB-dTAG dephosphorylation through the recruitment of bromoTAG-PPP2CA in an unbiased manner, we conducted quantitative phospho- and total-proteomic analyses in *TFEB*^*dTAG/dTAG*^*/*^*bromoTAG/+*^*PPP2CA* U2OS cells treated with vehicle (DMSO) or 100 nM BDPIC for 2 h (Figure 6). Total proteome analysis revealed a total of 8,127 proteins, of which only one protein, namely SSBP1, was observed to be significantly downregulated in abundance by more than 2-fold in BDPIC-treated cells compared to DMSO-treated controls (Figure 6B; Table 1). This suggests that BDPIC does not induce global proteomic changes in cells. A phospho-proteomic analysis following phospho-peptide enrichment in the two samples resulted in the identification of 30,278 unique phospho-peptides (Figure 6C). Of these, only 3 phospho-peptides corresponding to 3 proteins were significantly downregulated by > 1.5-fold in BDPIC-treated cells compared to DMSO-treated controls, while 5 phospho-peptides corresponding to 5 proteins were significantly upregulated by > 1.5-fold (Figure 6C; Tables 2 and 3). Notably, one of the significantly downregulated phospho-peptides detected in BDPIC-treated cells corresponded to was TFEB-pS455 (Figures 6C and 6D). One other TFEB phospho-peptide identified, pT330, was also significantly downregulated by ∼1.4-fold (Figures 6C and 6E). Because the quantitative phospho-proteomic analysis was performed in whole cell extracts, and not in TFEB-enriched samples, a lack of detection of several more phospho-TEFB peptides is not unexpected. Although beyond the scope of the current manuscript, prior enrichment of TFEB-dTAG via immunoprecipitation followed by quantitative phospho-proteomic analysis might reveal the full extent of targeted dephosphorylation by BDPIC in these cells.

**Table 1:** Downregulated peptides identified following global total proteome analysis of BDPIC-mediated TFEB-dTAG dephosphorylation

Protein	UniProt ID	Fold change	*p*-value
SSBP1	Q04837	0.406	0.022

Values of downregulated peptides identified (out of a total of 8,127 proteins detected) from global analysis of BDPIC-treated *TFEB*^*dTAG/dTAG*^*/*^*bromoTAG/+*^*PPP2CA* U2OS cells compared to DMSO-treated group. UniProt ID is indicated for the protein, as are corresponding peptides detected, fold changes and *p*-values. The threshold parameters included a significance level of *p* < 0.05 and fold change >2.

**Table 2:** Hypo-phosphorylated phospho-peptides identified following global phospho-proteomic analysis of BDPIC-mediated TFEB-dTAG dephosphorylation

Protein	UniProt ID	Hypo-phosphorylated phospho-peptides	Fold change	*p*-value
CEP152	O94986	HLNSLPR	0.689	0.008
DENND4B	O75064	GSPVPWHDGSLSDLSLTGEEPLPGG SPGGSGSALSAQSTEALEGLSGR	0.688	0.004
TFEB	P19484	DLDLMLLDDSLLPLASDPLLSTMSPEASK	0.667	0.012
		VHGLPTTSPSGMNMAELAQQVVK	0.791	0.033

Values of hypo-phosphorylated phospho-peptides identified (out of a total of 30,278 unique phospho-peptides detected) from global analysis of BDPIC-treated *TFEB*^*dTAG/dTAG*^*/*^*bromoTAG/+*^*PPP2CA* U2OS cells compared to DMSO-treated control group. UniProt ID is indicated for the protein, as are corresponding peptides detected, fold changes and *p*-values. The threshold parameters included a significance level of *p* < 0.05 and fold change>1.5. Phospho-peptides detected corresponding to TFEB are highlighted in green, including phospho-residues S455 and T330 (fold change below threshold) that were detected. Proteins are listed in alphabetical order.

**Table 3:** Hyper-phosphorylated phosphopeptides identified following global phosphoproteomic analysis of BDPIC-mediated TFEB-dTAG dephosphorylation

Protein	UniProt ID	Hyper-phosphorylated phospho-peptides	Fold change	*p*-value
ADAM22	Q9P0K1	SNSWQGNLGGNK	1.528	0.018
FBXW9	Q5XUX1	TWDDDSDPESETDPDAQAK	1.832	0.014
HECA	Q9UBI9	RHSMDRQNSQEK	1.504	0.024
LEO1	Q8WVC0	IQNSDDEERAQGSDEDK	1.645	0.050
LIN28B	Q6ZN17	VTGPGGSPCLGSER	1.611	0.025

Values of hyper-phosphorylated phospho-peptides identified (out of a total of 30,278 unique phospho-peptides detected) from global analysis of BDPIC-treated *TFEB*^*dTAG/dTAG*^*/*^*bromoTAG/+*^*PPP2CA* U2OS cells compared to DMSO-treated control group. UniProt ID is indicated for the protein, as are corresponding peptides detected, fold changes and *p*-values. The threshold parameters included a significance level of *p* < 0.05 and fold change>1.5. Proteins are listed in alphabetical order.

## Discussion

Targeted dephosphorylation of phospho-proteins in cells is an emerging modality. In this study, through the AdPhosphatase technology,^30^ we recruited the FLAG-aGFP_6M_-PPP1CA and FLAG-aGFP_6M_-PPP2CA to TFEB-GFP in *TFEB*^*GFP/GFP*^ knock-in cells to demonstrate phosphatase activity-dependent proof-of-concept for targeted dephosphorylation of TFEB. Protein phosphatase 1 (PP1) and protein phosphatase 2A (PP2A) are the two most ubiquitous serine/threonine phosphatases in eukaryotes and target thousands of substrates.^51^^,^^52^ Both PP1 and PP2A function as holoenzyme complexes consisting of multiple catalytic and regulatory subunits, with PPP1CA and PPP2CA thought to be their primary catalytic subunits, respectively.^51^^,^^53^^,^^54^ PP2A has been reported to trigger TFEB dephosphorylation in response to oxidative stress,^25^ even though the mechanism responsible for this remains to be elucidated. By employing the AdPhosphatase system, we were also able to target the dephosphorylation of TFEB to varying extents with multiple phosphatases, including calcineurin catalytic subunits PPP3CA-C, and protein phosphatase 5C, suggesting that different phosphatases can be redirected to dephosphorylate TFEB, potentially to achieve differing specificity and potency, through induced proximity. It would be interesting to test this possibility through an unbiased phospho-proteomic analysis in the future. The aGFP_6M_-PPP2CA AdPhosphatase-mediated dephosphorylation of TFEB-GFP was sufficient to cause its nuclear translocation. This is consistent with previous studies showing that phosphorylation of TFEB, for instance by mTORC1, leads to its retention in the cytosol, and a reduction in phosphorylation of TFEB via inhibition of mTORC1 or activation of AMPK pathway leads to nuclear accumulation of TFEB.^7^^,^^15^ Despite a robust nuclear accumulation of TFEB by the aGFP_6M_-PPP2CA AdPhosphatase, transcription of the selected TFEB-target genes appeared to be unaffected. Although we did not test this experimentally, this could be a limitation of the AdPhosphatase system, in which the constitutive binding of the aGFP_6M_-PPP2CA AdPhosphatase to TFEB-GFP at the transcription site might interfere with the assembly of transcription initiation complexes, thereby inhibiting transcription. The future use of the AdPhosphatase system should consider its constitutive binding to the target phospho-protein as a limitation that could have functional consequences, especially in cases such as transcription factors which rely on multi-protein complexes for biological activity. Alternatively, because the AdPhosphatase system caused dephosphorylation of TFEB to a greater extent than MK-8722 and Torin 1, it is possible that some phospho-residues critical for TFEB transcriptional activation may have been dephosphorylated leading to its inactivation.

We also developed the BDPIC induced-proximity platform to demonstrate chemical proof-of-concept for targeted dephosphorylation of TFEB. By using BDPIC, we demonstrated that bromoTAG-PPP2CA, knocked in at the native PPP2CA locus, can be redirected to dephosphorylate TFEB. This dephosphorylation was sufficient to trigger nuclear translocation of TFEB and transcription of *Gpnmb*, without the need for modulation of specific signaling pathways controlling TFEB. Strikingly, BDPIC action is transient and reversible, and can perform with great efficacy within the nM range. In a like-for-like comparison, BDPIC performed much better than other proximity-inducing bivalent molecules HDPIC and the previously reported PhosTAC7.^27^ Our findings demonstrate targeted dephosphorylation of TFEB as an effective approach to target nuclear translocation of TFEB and it could be considered as an innovative therapeutic approach in pathologies, such as metabolic disorders, where enhanced TFEB-dependent transcription leading to increased lysosomal and mitochondrial biogenesis in metabolic tissues such as muscle and liver may be beneficial. Our findings imply that by developing a phosphatase recruiting chimera (PhosTAC) molecule that connects to a POI on one end and another one to a phosphatase of interest could in principle be used for targeted dephosphorylation. A key limitation currently is the lack of small molecule binders of protein phosphatases that do not interfere with the phosphatase activities. A peptidic-binder of PP1 was coupled with an inhibitor of Akt to show a modest reduction in Akt phosphorylation,^26^ but peptidic ligands are typically large and are not cell-permeable. More recently, PP5-recruiting chimera molecule, DD03711, made with the activator of PP5, PP5SA-2, and ASK1 inhibitor, TCASK10, was shown to reduce the phosphorylation of ASK1 at T838.^28^ The specificity of the targeted dephosphorylation of ASK1 or the potential consequences of the activation of PP5 with DD03711 on its natural substrates were not tested.

The profound mobility shift observed for TFEB upon targeted dephosphorylation, which was more than that caused by pharmacological AMPK activation or mTORC1 inhibition, suggested that TFEB is phosphorylated at additional residues than those sites controlled by AMPK or mTORC1 pathways. Indeed, phosphoproteomic analysis have reported that there are more than 20 phosphorylated residues on TFEB^55^^,^^56^^,^^57^ in cells, however only few have been known to be linked to its transcriptional activity. The most well-studied mechanism that regulates TFEB phosphorylation is through mTORC1.^16^^,^^17^^,^^18^^,^^19^^,^^20^ Three main sites, S122, S142 and S211, of TFEB are reported to be phosphorylated by mTORC1, of which S211 has been shown to be critical for cytoplasmic retention of TFEB.^17^ Even though several other kinases have been proposed to phosphorylate TFEB *in vitro* or in cells, including ERK2 (S142), GSK3β (S134 and S138), PKCβ (S462, S463, S467 and S469), Akt (S467) and AMPK (S466, S467 and S469), which are reported to alter subcellular distribution or transcriptional activity of TFEB, conclusive evidence for their functional consequences remains elusive.^22^^,^^58^^,^^59^ Targeted dephosphorylation of specific phospho-residues on TFEB, if it could be achieved, could help dissect the function of TFEB phosphorylation on specific residues. By phospho-proteomic analysis, we identified several phospho-peptides in *TFEB*^*GFP/GFP*^ knock-in C2C12 cells that were absent upon targeted dephosphorylation by aGFP_6M_-PPP2CA AdPhosphatase. However, due to the low abundance of TFEB-GFP in *TFEB*^*GFP/GFP*^ knock-in C2C12 cells, we were unable to detect the full atlas of reported TFEB phospho-peptides. Similarly, by unbiased global quantitative phosphoproteomic analysis of BDPIC-induced dephosphorylation in *TFEB*^*dTAG/dTAG*^*/*^*bromoTAG/+*^*PPP2CA* U2OS cells, we were able to detect only two phospho-peptides on TFEB (pT330 and pS455) and both were significantly downregulated by BDPIC treatment compared to DMSO control. Although the global phospho-proteomic analysis did not identify other TFEB phospho-peptides, the coverage of more TFEB phospho-peptides could be improved in the future by enriching TFEB by immunoprecipitation prior to phospho-proteomic analysis. Nonetheless, it was reassuring that BDPIC only resulted in very few total proteomic and phospho-proteomic changes, suggesting minimal off-target effects of BDPIC and not many consequences of redirecting PPP2CA on the phosphorylation levels of native PPP2CA substrates. In this regard, a comprehensive list of over 2000 potential PPP2CA substrates was reported recently.^50^

BDPIC is an efficient proximity inducer between dTAG and bromoTAG. We have shown its utility in targeted dephosphorylation through induced proximity between bromoTAG-PPP2CA and TFEB-dTAG here, as well as dTAG-SMAD3 and bromoTAG-PPM1H.^60^ In principle, BDPIC could be applied to induce proximity between any two proteins inside cells. The molecule offers an excellent platform to test whether modulating any of the more than 500 protein post-translational modifications on target proteins by recruiting the modifying enzymes in proximity affects the target protein function. Such approaches could potentially reveal innovative drug modalities, as we have done here with targeted dephosphorylation. As we have shown here, the CRISPR/Cas9 gene editing technology allows for relatively efficient knock-ins of dTAG and bromoTAG onto target proteins and enzymes of interest in any cell type, provided the tags do not compromise the function of the proteins. Given there are PROTACs directed at these tags, protein or enzyme loss of function studies through targeted protein degradation can also be investigated.

### Limitations of the study

One key limitation of the AdPhosphatase approach is its constitutive binding to the target protein, which might cause functional alteration of the target protein regardless of targeted dephosphorylation. Since BDPIC was synthesized only with a single 3-PEG linker, testing more compounds with alternative linkers may improve the compound efficacy and potentially allow targeted dephosphorylation of different set of phospho-sites. We showed that in *TFEB*^*dTAG/dTAG*^*/*^*bromoTAG/+*^*PPP2CA U2OS* cells BDPIC causes negligible off-target effects in terms of changes in global phospho proteome. Nonetheless, we note that the current study lacks systematic characterization, including proteomic, phospho-proteomic and transcriptomic analyses in both parental and knock-in cells with different treatment kinetics, to address all potential off-target effects of BDPIC. The current approach requires addition of bromoTAG or dTAG to the phospho-POIs and phosphatases in the same cells for BDPIC-mediated targeted dephosphorylation. Therefore, the potential impact of introducing these tags on the POI and phosphatase function needs to be considered and tested. We did not assess site-specific dephosphorylation of TFEB in this study. However, the fact that different phosphatases appear to cause varying levels of TFEB-dTAG mobility shifts implies unique site-specific dephosphorylation of TFEB could be achieved simply by recruiting different phosphatases. To validate this hypothesis, we would require robust quantitative phospho-proteomic analysis pipeline. The differences observed on the extent of BDPIC-induced targeted dephosphorylation of TFEB-dTAG between overexpressed bromoTAG-PPP2CA in *TFEB*^*dTAG/dTAG*^ U2OS cells *vs.* endogenous bromoTAG-PPP2CA in *TFEB*^*dTAG/dTAG*^*/*^*bromoTAG/+*^*PPP2CA* U2OS cells suggest that the abundance of the phosphatase, and therefore the stoichiometry of the resulting ternary complex upon BDPIC treatment, may impact the level of targeted dephosphorylation of the target protein. This study lacks the full scope of physiological implications of targeted TFEB dephosphorylation and follow-up studies using *in vivo* models are needed to understand the biological significance of the targeted dephosphorylation of TFEB.

## STAR★Methods

### Key resources table

**Table:** 

REAGENT or RESOURCE	SOURCE	IDENTIFIER
**Antibodies**		

Rabbit monoclonal anti-TFEB p-S211	Cell Signaling Technology	Cat#37681
Rabbit monoclonal anti-TFEB p-S122	Cell Signaling Technology	Cat#87932
Rabbit polyclonal anti-TFEB p-S142	Merck	Cat#ABE1971-I
Rabbit polyclonal anti-TFEB p-S109	This paper	N/A
Rabbit polyclonal anti-TFEB p-S138	This paper	N/A
Rabbit polyclonal TFEB	ProteinTech	Cat#13372-1-AP
GAPDH Monoclonal antibody	ProteinTech	Cat#60004-1-Ig
Rat monoclonal anti-alpha-tubulin	Invitrogen	Cat#MA1-80189
Mouse monoclonal anti-beta-actin	ProteinTech	Cat#66009-1
FLAG M2-Peroxidase (HRP) mouse monoclonal antibody	Sigma	Cat#A8592
Rabbit polyclonal anti-GFP	MBL	Cat#598
Sheep polyclonal anti-GFP	MRC PPU Reagents & Services	Cat#S268B
Sheep polyclonal anti-PP2CA/B	MRC PPU Reagents & Services	Cat#S274B
Sheep polyclonal anti-dTAG	MRC PPU Reagents & Services	Cat#DA179
Sheep polyclonal anti-bromoTAG	MRC PPU Reagents & Services	Cat#DA599
goat anti-Rabbit IgG (H + L)-HRP	Cell Signaling Technology	Cat#7074
horse anti-mouse IgG (H + L)-HRP	Cell Signaling Technology	Cat#7076
Goat anti-Rat IgG (H + L)-HRP	Thermo Fisher Scientific	Cat#31470
rabbit anti-sheep IgG (H + L)-HRP	Thermo Fisher Scientific	Cat#31480

**Chemicals, peptides, and recombinant proteins**		

MK-8722	MedChemExpress	Cat#HY-111363
Torin 1	Tocris	Cat#4247
BDPIC	Synthesized by Natalia Shpiro as described in this manuscript	N/A
dTAG^V^-1-NEG	Synthesized by Natalia Shpiro as described in this manuscript	N/A
*cis*-AGB1	Synthesized by Natalia Shpiro as described in this manuscript	N/A
HDPIC	Synthesized by Natalia Shpiro as described in this manuscript	N/A
PhosTAC7	Synthesized by Natalia Shpiro as described in this manuscript	N/A
ANTI-FLAG M2 Affinity Gel	Sigma-Aldrich	Cat#A2220
ProLong Gold Antifade Mountant with DAPI	Life Technologies	Cat#P36935
PEI MAX – Transfection Grade Linear PEI Hydrochloride MW 40,000	Polysciences	Cat#24765
Polybrene (Hexadimethrine bromide)	Sigma-Aldrich	Cat#107689
Lambda Protein Phosphatase	New England Biolabs	Cat#P7053S
Dimethylsulphoxide	Sigma-Aldrich	Cat#D8418
Formic acid (FA)	Sigma-Aldrich	Cat#F0507
Trifluoroacetic acid	Sigma-Aldrich	Cat #T6508-100ML
Triethylammonium bicarbonate buffer (TEAB)	Thermo Fisher Scientific	Cat#PI90114
Dithiothreitol	Sigma-Aldrich	Cat#D0632
Iodoacetamide (IAA)	Sigma-Aldrich	Cat#I6125
Trypsin	Promega	Cat#V5111
Lys-C	Wako	Cat#125–05061
Acclaim PepMap 100 2 cm trap column	Thermo Fisher Scientific	N/A
Acclaim PepMap 100C18 HPLC Column, 50 cm	Thermo Fisher Scientific	N/A
PhosStop phosphatase inhibitor cocktail tablets	Roche	Cat#4906837001
Acetonitrile	JT Baker	Cat#14650359

**Critical commercial assays**		

Pierce BCA Protein Assay Kit	Thermo Fisher Scientific	Cat#23225
TMTsixplex Isobaric Label Reagent Set	Thermo Fisher Scientific	Cat#90066
alamarBlue™ HS Cell Viability Reagent	Thermo Fisher Scientific	Cat#A50100
PowerUp SYBR green Master mix	Thermo Fisher Scientific	Cat#A25777

**Deposited data**		

Phosphoproteomics	This paper	PRIDE Project ID: PXD053308
Proteomics	This paper	PRIDE Project ID: PXD053311
Data obtained in this study	This paper	Mendeley Data: https://data.mendeley.com/datasets/mfmmy5dbb2/1

**Experimental models: Cell lines**		

Human: HEK293-FT	Invitrogen	Cat# R70007
Human: U2OS	ATCC	Cat# HTB-96
Mouse: C2C12	ATCC	Cat# CRL-1772

**Recombinant DNA**		

pCMV5-gag-pol	Cell Biolabs	Cat# RV-111
pCMV5-VSV-G	Cell Biolabs	Cat# RV-110
pBabeD-puromycin FLAG-aGFP_6M_	MRC PPU Reagents & Services	DU60421
pBabeD-puromycin FLAG-aGFP_6M_-PPP1CA	MRC PPU Reagents & Services	DU62917
pBabeD-puromycin FLAG-aGFP_6M_-PPP1CA^H125Q^	MRC PPU Reagents & Services	DU62964
pBabeD-puromycin FLAG-aGFP_6M_-PPP2CA	MRC PPU Reagents & Services	DU62902
pBabeD-puromycin FLAG-aGFP_6M_-PPP2CA^H118Q^	MRC PPU Reagents & Services	DU62960
pBabeD-puromycin FLAG-aGFP_6M_-PPP3CA	MRC PPU Reagents & Services	DU78402
pBabeD-puromycin FLAG-aGFP_6M_-PPP3CA^H151Q^	MRC PPU Reagents & Services	DU78403
pBabeD-puromycin FLAG-aGFP_6M_-PPP3CB	MRC PPU Reagents & Services	DU78406
pBabeD-puromycin FLAG-aGFP_6M_-PPP3CB^H160Q^	MRC PPU Reagents & Services	DU78407
pBabeD-puromycin FLAG-aGFP_6M_-PPP3CC	MRC PPU Reagents & Services	DU78408
pBabeD-puromycin FLAG-aGFP_6M_-PPP3CC^H147Q^	MRC PPU Reagents & Services	DU78409
pBabeD-puromycin FLAG-aGFP_6M_-PPP5C	MRC PPU Reagents & Services	DU78398
pBabeD-puromycin FLAG-aGFP_6M_-PPP5C^H304A^	MRC PPU Reagents & Services	DU78399
pBabeD-puromycin 3HA-5Gly-Bromotag(L387A)-5Gly	MRC PPU Reagents & Services	DU71673
pBabeD-puromycin 3HA-5Gly-Bromotag(L387A)-5Gly-PPP2CA	MRC PPU Reagents & Services	DU75905
pBabeD-puromycin 3HA-5Gly-Bromotag(L387A)-5Gly-PPP2CA^H118Q^	MRC PPU Reagents & Services	DU75906
pBabeD-puromycin 3HA-5Gly-Bromotag(L387A)-5Gly-PPP3CA	MRC PPU Reagents & Services	DU78400
pBabeD-puromycin 3HA-5Gly-Bromotag(L387A)-5Gly-PPP3CA^H151Q^	MRC PPU Reagents & Services	DU78401
pBabeD-puromycin 3HA-5Gly-Bromotag(L387A)-5Gly-PPP3CB	MRC PPU Reagents & Services	DU78404
pBabeD-puromycin 3HA-5Gly-Bromotag(L387A)-5Gly-PPP3CB^H160Q^	MRC PPU Reagents & Services	DU78405
pBabeD-puromycin 3HA-5Gly-Bromotag(L387A)-5Gly-PPP5C	MRC PPU Reagents & Services	DU78396
pBabeD-puromycin 3HA-5Gly-Bromotag(L387A)-5Gly-PPP5C^H304A^	MRC PPU Reagents & Services	DU78397

**Software and algorithms**		

Proteome Discoverer platform (v2.4)	Thermo Fischer Scientific	NA
Image Lab	Bio-Rad	https://www.bio-rad.com/en-uk/product/image-lab-software?ID=KRE6P5E8Z
GraphPad Prism v9.4.0	GraphPad	https://www.graphpad.com/scientific-software/prism/

### Resource availability

#### Lead contact

Further information and requests for resources and reagents should be directed to and will be fulfilled by the Lead Contact, Gopal Sapkota (g.sapkota@dundee.ac.uk).

#### Materials availability

All constructs used in this study are available to request from the MRC PPU Reagents & Services webpage (http://mrcppureagents.dundee.ac.uk) and the unique identifier (DU) numbers provide direct links to the cloning strategies and sequence details. All constructs were sequence-verified by the DNA Sequencing Service, University of Dundee (http://www.dnaseq.co.uk).

#### Data and code availability

The datasets generated during this study are publicly available at Mendeley Data (https://data.mendeley.com/datasets/mfmmy5dbb2/1). The link to access these data can also be found in the key resources table. All raw mass spectrometry data acquired from this study has been deposited in the PRIDE Archive, with the accession number PXD053308 (Phospho-proteomics) and PXD053311 (Total proteomics). This paper does not report original code. Any additional information required to reanalyse the data reported in this paper is available from the lead contact upon request.

### Experimental model and study participant details

#### Cell lines

Mouse myoblast C2C12 (ATCC, Cat# CRL-1772), human embryonal kidney cells 293FT (Invitrogen, Cat# R70007) and human osteosarcoma U2OS (ATCC, Cat# HTB-96) cells were cultured in DMEM (Life Technologies) with 10% FBS (Thermo Fisher Scientific), 2 mM L-glutamine (Lonza) and 1% streptomycin/penicillin (Lonza). All cell lines were maintained at 37°C and under 5% CO_2_ in a humidified tissue culture incubator. All cell lines were verified to be free of mycoplasma contamination before conducting any experiment. Cells were exposed to different stimuli and compounds as described in the appropriate figure legends prior to lysis.

### Method details

#### Materials

Rabbit anti-phospho-TFEB Ser109 and anti-phospho-TFEB Ser138 were custom generated via YenZym by immunizing rabbits with the following antigens: for phospho-TFEB Ser109 - human TFEB peptide incorporating residues 102–113 (NKFAAHI-pS-PAQG) with pS at 109; for phospho-TFEB Ser138 - human TFEB peptide incorporating residues 128–140 (GHVLSSSAGN-pS-AP) with pS at 138. The antibodies were affinity purified using the phospho-peptide antigens and further cleared through the corresponding immobilized dephospho-peptides to remove any non-phospho binding. These antibodies were tested for specificity by immunoblotting using HEK293 extracts expressing TFEB WT, S109A and S138A mutants (Figures S10B and S10C).

#### Plasmids

For production of retroviral vectors, the following were cloned into pBABED-puromycin plasmids: FLAG-aGFP_6M_-PPP1CA (DU62917), FLAG-aGFP_6M_-PPP1CA^H125Q^ (DU62964), FLAG-aGFP_6M_-PPP2CA (DU62902), FLAG-aGFP_6M_-PPP2CA^H118Q^ (DU62960), FLAG-aGFP_6M_-PPP3CA (DU78402), FLAG-aGFP_6M_-PPP3CA^H151Q^ (DU78403), FLAG-aGFP_6M_-PPP3CB (DU78406), FLAG-aGFP_6M_-PPP3CB^H160Q^ (DU78407), FLAG-aGFP_6M_-PPP3CC (DU78408), FLAG-aGFP_6M_-PPP3CC^H147Q^ (DU78409), FLAG-aGFP_6M_-PP5C (DU78398), FLAG-aGFP_6M_-PP5C^H304A^ (DU78399), 3HA-5Gly-bromoTAG-5Gly (DU71673), 3HA-5Gly-bromoTAG-5Gly-PPP2CA (DU75905), 3HA-5Gly-bromoTAG-5Gly-PPP2CA^H118Q^ (DU75906), 3HA-5Gly-bromoTAG-5Gly-PPP3CA (DU78400), 3HA-5Gly-bromoTAG-5Gly-PPP3CA^H151Q^ (DU78401), 3HA-5Gly-bromoTAG-5Gly-PPP3CB (DU78404), 3HA-5Gly-bromoTAG-5Gly-PPP3CB^H160Q^ (DU78405), 3HA-5Gly-bromoTAG-5Gly-PP5C (DU78396), 3HA-5Gly-bromoTAG-5Gly-PP5C^H304A^ (DU78397). All constructs were sequence-verified by the DNA Sequencing Service, University of Dundee (http://www.dnaseq.co.uk). For CRISPR/Cas9 gene editing, the following guide RNAs (gRNA) and donor cDNAs were used: mouse *TFEB* C-terminal GFP knockin (KI): sense gRNA (DU69664), antisense gRNA (DU69669), GFP donor (69673). Mouse *TFE3* konckout: sense gRNA (DU69717), antisense gRNA (DU69720). Human *TFEB* C-terminal dTAG KI: single gRNA (DU69426), dTAG-IRES2-GFP donor (DU74451). Human *PPP2CA* N-terminal bromoTAG KI: single guide (DU69331), mCherry-IRES2-bromoTAG donor (DU74520). These constructs are available to request from the MRC-PPU Reagents and Services webpage (http://mrcppureagents.dundee.ac.uk) and the unique identifier (DU) numbers also provide direct links to the cloning strategies and sequence details.

#### Generation of cell lines using CRISPR/Cas9

The CRISPR/Cas9 genome editing system^61^ was used to generate KI and KO cells. To generate mouse TFEB-GFP KI cells, C2C12 cells were transfected with vectors encoding a pair of guide RNAs (pBabeD-puro-sgRNA1 (1 μg) and pX335-CAS9-D10A-sgRNA2 (1 μg)) targeting close to the stop codon of *TFEB*, along with the respective donor plasmid carrying the in-frame GFP KI insert and flanking *TFEB* homology arms (3 μg). For the generation of mouse TFE3 KO cells, C2C12 *TFEB*^*GFP/GFP*^ cells were transfected with vectors encoding a pair of guide RNAs (pBABED-Puro-sgRNA1 (1 μg) and pX335-CAS9-D10A-sgRNA2 (1 μg)) targeting on the exon 3 of *TFE3*. To generate human TFEB-dTAG KI cells, U2OS cells were transfected with vectors encoding a single gRNA (pX459-CAS9-gRNA (1 μg)) targeting close to the stop codon of *TFEB* (*TFEB*^*dTAG/dTAG*^) and donor plasmid carrying the dTAG-IRES2-GFP KI cassette flanked by *TFEB* homology arms (3 μg). For the generation of human bromoTAG-PPP2CA KI cells, U2OS *TFEB*^*dTAG/dTAG*^ cells were transfected with vectors encoding a single gRNA (pX459-Puro-CAS9-gRNA (1 μg)) targeting on the N-terminal of PPP2CA (to express bromoTAG-PPP2CA) and the donor plasmid carrying mCherry-IRES2-bromoTAG KI cassette flanked by *PPP2CA* homology arms (3 μg). One day post-transfection, cells were selected with 2 μg/mL puromycin for a further 48 h. For KO cells, cells were expanded in fresh medium before western blotting analysis to confirm knockdown efficiency. For KI cells, the transfection process was repeated one more time when the cell confluency reached 60–70% following selection in puromycin. 24 h post-transfection, cells were maintained in fresh medium until single cell isolation. For the acquisition of single-cell clones of KOs, and GFP, dTAG and bromoTAG KIs, single cells were isolated by fluorescence-activated cell sorting (FACS) using an Influx cell sorter (Becton Dickinson). Single cell clones were plated on individual wells of 96-well plates, pre-coated with 1% (w/v) gelatin to help with cell adherence. Viable clones were expanded, and successful KO or integration of GFP, dTAG or bromoTAG cDNA at the target locus was confirmed by both Western blotting and genomic DNA sequencing.

#### Retroviral generation of stable cell lines

Stable cell lines were generated by retroviral transduction. The cDNA of interest was inserted into a pBabeD-puromycin vector (6 μg) and co-transfected with pCMV5-GAG/POL (3.8 μg) and pCMV5-VSV-G plasmids (2.2 μg) (Clontech) into a 10-cm dish of ∼60% confluent 293FT cells by using 36 μL of 1 mg/mL PEI. 24h post-transfection, fresh medium was added into the cells. The medium containing the retroviral particles was harvested 48 h post-transfection, passed through a 0.45 mm filter and added to target cells in the presence of 10 μg/mL polybrene (Sigma-Aldrich). Cells were selected with 2 μg/mL puromycin (Sigma-Aldrich) for 48 h after exposure to retroviral particles. A pool of transduced cells was utilized for subsequent experiments following selection.

#### Cell lysis

Cells were harvested by washing twice with phosphate-buffered saline (PBS) and scraping into ice-cold lysis buffer containing 50 mM HEPES pH7.4, 150 mM NaCl, 1 mM EDTA, 10% glycerol, 0.5% NP-40, 1 mM DTT, 1 mM PMSF, 1.15 mM sodium molybdate, 4 mM sodium tartrate, 10 mM β-glycerophosphate, 1 mM sodium fluoride, 1 mM sodium orthovanadate and 1x complete protease inhibitor cocktail (Roche). After incubation for 10 min on ice, lysates were cleared by centrifugation at 20,000 x g for 10 min at 4°C. Protein concentration was determined using Bradford protein assay. For chemical cross-linking by DSP, cells were treated with compounds for 2 h before lysis. For this, after washing cells with pre-warmed PBS twice, cells were incubated with pre-warmed PBS containing 1 mM DSP and compounds for further 30 min at 37°C. The reaction was quenched by adding 20 mM Tris-HCl in PBS for 20 min. Cells were then lysed in lysis buffer without reducing agents.

#### Immunoprecipitation

Following determination of protein concentration by Bradford assay, immunoprecipitation (IP) was performed to isolate a particular protein of interest. For anti-FLAG IPs, anti-FLAG M2 resin (Sigma-Aldrich) was used; for anti-HA IP, anti-HA frankenbody Sepharose beads (MRC-PPU Reagents and Services) was used. Before an IP was performed, a small amount of each lysate (10–25 μg protein) was retained to compare and determine IP efficiency. Samples (1 mg total protein) were incubated with 10 μL packed beads overnight at 4°C on a rotating wheel. Beads were collected by centrifugation at 1000 x *g* for 5 min at 4°C and a sample of the supernatant was retained for post-IP flowthrough extract control. IPs were subsequently washed twice with 1 mL lysis buffer in the presence of 500 mM NaCl, once in 1 mL of lysis buffer and then resuspended in 20 μL of lysis buffer. Input, IP and post-IP flowthrough extract (equivalent to input) samples were reduced in 1X LDS sample buffer (Invitrogen) and boiled at 95°C for 5 min prior to SDS-PAGE.

#### Immunoblotting

Reduced and denatured cell lysates containing equal amounts of protein (10–25 μg) were resolved by SDS-PAGE and transferred to nitrocellulose membranes. After blocking in 5% (w/v) non-fat milk in TBS-T (20 mM Tris, 150 mM NaCl, 0.1% Tween-20) for 1 h at room temperature, membranes were incubated with primary antibody diluted in 5% milk TBS-T overnight at 4°C in a shaker. Membranes were then washed 3 × 10 min in TBS-T with constant shaking and subsequently incubated with HRP-conjugated secondary antibody diluted in 5% milk TBS-T solution for 1 h at room temperature. Membranes were then washed 3 × 10 min in TBS-T and the signal detection via chemiluminescence (Promega) was performed using ChemiDoc imaging system (Bio-Rad).

#### Immunofluorescence microscopy

Cells were seeded onto sterile glass coverslips in 12-well dishes. After treatments, cells were washed twice with PBS and fixed with pre-chilled methanol for 20 min. The cells were blocked by washing twice and incubation for 30 min in blocking buffer (1% (w/v) BSA/PBS). Coverslips were incubated for 1 h at 37°C with primary antibodies in blocking buffer and washed three times in blocking buffer. Coverslips were then incubated for 1 h at room temperature with Alexa Fluor coupled secondary antibodies (Life Technologies) in blocking buffer and washed an additional three times in blocking buffer. After submerging in ddH_2_O, cells were mounted onto glass slides using prolong gold antifade mountant containing DAPI (Life Technologies) and visualized with a Zeiss LSM880 Airyscan or LSM710 Confocal Scanning microscope (ZEISS; Plan Apochromat X63 objective, NA 1.4). Images were processed using ZEISS Zen Software.

#### Quantitative reverse transcription PCR (RT-qPCR)

Total cellular RNA was isolated from C2C12 or U2OS cells using a RNeasy Micro Kit (Qiagen) according to the manufacturer’s instructions. 1 μg total RNA was converted to cDNA by using SuperScript cDNA kit. The obtained cDNA samples were used as templates for qPCR by CFX384 real-time qPCR machine (Bio-Rad). Data was normalized with the mean of housekeeping gene *Tbp*. The following primer sequences were used: mouse *Hexa*: sense 5′- GCT GAG GGC ACG TTC TTT ATC-3′, antisense 5′- GCG AGA TGT ATC CAG CAG TAC G-3’; mouse *Fnip1*: sense 5′- GAT GCG TGT TCA TGT CAA GG-3′, antisense 5′- GGA GAG TGG GTG CTT GCT AC-3’; mouse *Flcn*: sense 5′- TGG ATC GGA TCT ACC TCA TCA-3′, antisense 5′- TGG ACA TCC AAA CTG CTC TG-3’; mouse *Tbp*: sense 5′-CCT TGT ACC CTT CAC CAA TGA C -3′, antisense 5′-ACA GCC AAG ATT CAC GGT AGA -3’; human *Hexa*: sense 5′- CAA CCA ACA CAT TCT TCT CCA-3′, antisense 5′- CGC TAT CGT GAC CTG CTT TT; human *Fnip1*: sense 5′- GGT TCT CGG TGC TCT TCT GAT-3′, antisense 5′- GCT GTG GAG GGG AAC GAA T-3’; human *Flcn*: sense 5′- GAT TGA AGC GGC TCT GAC CAA C-3′, antisense 5′- TCG ACT GTC CAC CTT GGT GAA C-3’; human *Gpnmb* sense 5′- GAT GCC AAA AGG AAG ATG CC-3′, antisense 5′- CTC TGA CCA TGC TGT CCA GTT-3’; human *Tbp*: sense 5′- AGG GTT TCT GGT TTG CCA AGA-3′, antisense 5′- CTG AAT AGG CTG TGG GGT CA-3’.

#### Cell viability assay

0.5X10^4^ cells were plated onto each well of 96-well plate overnight and incubated in a humidified incubator with 5% CO_2_ at 37°C. BDPIC compound with various concentrations were added to culture medium for another 48 h. Following incubation, 10 μL of alamarBlue reagent (Thermo) in an amount equal to 10% of the total volume were added into each well and incubated for 2 h in a culture incubator at 37°C. Absorbance at 570 nm and 600 nm wavelengths was measured.

#### Immunoprecipitation followed by mass spectrometry

Cells were first lysed in NP-40 lysis buffer. Clarified lysates (5 mg protein) were incubated with Flag resin (25 μL packed beads) for 4 h on a rotating wheel at 4°C. Following incubation, beads were washed 3x with standard lysis buffer. Bead-bound proteins were denatured and eluted in 2x LDS for 5 min at 95°C. Samples were then filtered through Spin-X columns to remove the beads from the eluate. The filtered eluate was loaded onto a 4–12% Bis-Tris gradient gel and proteins were separated by SDS-PAGE. Gels were stained with InstantBlue and subsequently de-stained in deionised water. A small portion of the eluate was retained for analysis and validation by Western blotting. To minimise potential protein contaminants, all steps from this point were performed under a laminar flow hood. Disposable scalpels were used to cut protein bands of interest from the InstantBlue stained gels into 1–2 cm cubes, which were subsequently transferred into LoBind 1.5 mL Eppendorf tubes. Gel pieces were washed once in HPLC grade water, and then shrank in anhydrous acetonitrile (ACN) for 5 min with gentle shaking. The ACN was aspirated, and gel pieces were re-swollen with 50 mM Tris-HCl pH 8.0 for 5 min with shaking. The shrinking-swelling process was repeated once more, and the proteins within the gel pieces were reduced with 5 mM DTT in 50 mM Tris-HCl pH 8.0 for 20 min at 65°C. Next, the proteins within the gel pieces were alkylated with 20 mM iodoacetamide (IAA) in 50 mM Tris-HCl pH 8.0 for 20 min at room temperature in the dark. Gel pieces were then shrunk again in can for 5 min, dried and re-swollen in 50 μL of 50 mM triethylammonium bicarbonate (TEAB) pH 8.0 containing 5 mg/mL trypsin. After removing excess trypsin, gel pieces were covered in 50 mM TEAB and samples incubated in a shaker overnight at 37°C for tryptic digestion. An equivalent volume of ACN was added to the digest for 15 min with shaking and the supernatant was collected into a fresh LoBind 1.5 mL Eppendorf tube. Gel pieces were then re-swollen with 0.1% (v/v) trifluoroacetic acid (TFA) for 5 min with shaking, and peptides were extracted twice with ACN for 5 min each with shaking. After each extraction, the supernatant was collected and combined with the previous supernatants. The supernatants were then dried by vacuum centrifugation using a SpeedVac. Digested peptides were reconstituted in HPLC-grade 5% (v/v) ACN containing 0.1% (v/v) formic acid (FA) and into Orbitrap Fusion Tribrid mass spectrometer interfaced with Dionex Ultimate 3000 nanoflow liquid chromatography system. Peptides were trapped on a nanoViper Trap column (Acclaim PepMap 100, C18, 100 μm × 2 cm, 5 μm, 100 Å, Thermo Fisher Scientific) and subsequently separated on a 15-cm EasySpray column (PepMap RSLC C18, 75 μm × 50 cm, 2 μm, 100 Å75 μm × 50 cm, RSLC C18) equilibrated with a flow rate of 300 nL/min. Data was acquired in the data-dependent mode, automatically switching between MS1 and MS2 acquisition. Full scan spectra (m/z 375-1,500) were acquired in the orbitrap with resolution set to 120,000 at m/z 400. The 10 most intense ions, above a specified minimum signal threshold of 5,000, were fragmented by high collision induced dissociation. For MS analysis, a full automatic gain control (AGC) target of 400000 and ion injection time 50 ms were employed. For MS2 analysis, AGC target 1000 and ion injection time of 50 ms were used. Raw files were subsequently converted into a list of identified peptides, along with the precursor intensity of the identified peptides, and submitted to the in-house Mascot server (MRC-PPU, University of Dundee). Data was searched against the SwissProt human database with variable modifications allowing for oxidation of Met, phosphorylation of Ser/Thr or Tyr residues, along with oxidation or dioxidation modifications. Carbamidomethylation of Cys was set as a fixed modification. Error tolerances were set to 10 ppm (parts per million) for MS1 and 0.02 Da for MS2. Data analysis was performed using Scaffold v 4.4.6 (Proteome Software).

#### Global proteome and phospho-proteome analysis

Cells were treated with 100 nM BDPIC for 2 h in 5 biological replicates for global proteome and phospho-proteome analysis. The targeted dephosphorylation of TFEB by molecular weight shifts were confirmed by western blot prior to processing samples for proteomic analysis. Cells were lysed in urea lysis buffer (8 M urea, 50 mM Triethylammonium bicarbonate buffer (TEAB) pH 8.0, supplemented with cOmplete protease inhibitors and 1 tablet of PhosSTOP phosphatase inhibitors) by sonication (40% amplitude; on/off 10 s × 10 cycles) in LoBind Eppendorf tubes. Lysates were clarified by centrifugation for 20 min at 13,000 g at 4°C. Protein concentration was estimated using the Pierce bicinchoninic acid method. Equal protein from each condition was reduced with 5 mM dithiothreitol (DTT) at room temperature for 30 min and alkylated with 20 mM iodoacetamide (IAA) in the dark at room temperature for 15 min. Samples were then diluted with 50 mM Triethylammonium bicarbonate buffer (TEAB) to a urea concentration of less than 2M and were then digested with trypsin (1:20) at 30°C for 16 h. The digestion was quenched with the addition of trifluoroacetic acid (TFA) to give a final concentration of 1% TFA (v/v) and samples were desalted on 200 mg Sep-Pak C18 cartridges (Waters). For Sep-Pak clean-up, the following solvents were prepared fresh: activation solvent (100% (v/v) acetonitrile (ACN)); Solvent-1 (0.1% (v/v) TFA); Solvent-2 (0.1% (v/v) formic acid (FA)); Solvent-3 (50% (v/v) ACN in 0.1% (v/v) FA). Sep-Pak cartridges were equilibrated with 5 mL 100% ACN, followed by 5 mL 50% ACN, 0.1% FA and finally with 5 mL 0.1% TFA twice. Samples were then loaded onto the equilibrated C18 cartridges, washed with 5 mL 0.1% TFA four times, followed by washing with 5 mL 0.1% FA. Samples were then eluted with 1 mL 50% ACN, 0.1% FA. Desalted samples were then dried to completeness in a SpeedVac concentrator. Peptides were dissolved in 100 mM TEAB and labeled using TMT labels as per the manufacturer’s instructions. TMT labels were resuspended in anhydrous acetonitrile, added to assigned samples and incubated for 1 h at room temperature. The labeling reaction was quenched with 5% hydroxylamine for 15 min at room temperature. Labeled peptides from each condition were pooled together and dried. Pooled peptides were resolved by basic reverse phase chromatography fractionation on a C18, 250 × 4.6 mm column, 5 μm, XBridge (Waters, Milford, MA) with flow rate at 500 μL/min with two buffers: buffer A (10 mM ammonium formate, pH 10) and buffer B (80% ACN, 10 mM ammonium formate, pH 10). Peptides were dissolved in 120 μL of buffer A (10 mM ammonium formate, pH10) and separated on a C18 reverse phase column by applying a non-linear gradient of 7–40%. A total of 96 fractions were collected and concentrated into 24 fractions for proteomics analysis. 90% sample was used for immobilized metal affinity chromatography (IMAC)-based phospho-peptide enrichment and pooled in to 12 fractions. Each concentrated fraction was then dried by SpeedVac. IMAC beads were prepared from Ni-NTA (nitrilotriacetic acid) superflow agarose beads that were stripped of nickel with 100 mM EDTA and incubated in an aqueous solution of 10 mM iron (III) chloride (FeCl3) to prepare Fe-IMAC beads. Dried peptide fractions were dissolved to a concentration of 0.5 μg/μL in 80% ACN/0.1% TFA. Mixture of peptides were incubated with beads for 30 min with end-to-end rotation. Stage tips were prepared by equilibrating with methanol followed by 50% ACN/0.1% FA then 1% FA. The beads with enriched peptides were loaded onto C18 stage tips and washed with 80% ACN/0.1% TFA. Phosphorylated peptides were eluted from IMAC beads with 500 mM dibasic sodium phosphate, pH 7.0. These peptides were washed with 1% FA before elution using 50% acetonitrile in 0.1% FA. The peptides were then dried by SpeedVac and stored at −80°C until mass spectrometry analysis. Enriched phospho-peptides and peptides were analyzed on an Orbitrap Fusion Tribrid mass spectrometer interfaced with Dionex Ultimate 3000 nanoflow liquid chromatography system. For proteomic analysis, peptides were resolved on an analytical column (Acclaim PepMap RSLC C18, 75 μm × 50 cm, 2 μm, 100 Å) at a flow rate of 300 nL/min, using a step gradient of 5–7% solvent B (90% ACN/0.1% FA) for the first 10 min, followed by 7–25% up to 70 min and 25–35% up to 70–85 min. The total run time was set to 100 min. The mass spectrometer was operated in a data-dependent acquisition mode in SPS MS3 (FT-IT-HCD-FT-HCD) method. A survey full scan MS (from m/z 400–1400) was acquired in the Orbitrap at a resolution of 120,000 at 200 m/z. The AGC target for MS1 was set as 4 × 105 and ion filling time as 50 ms. The precursor ions for MS2 were isolated using a Quadrupole mass filter at a 0.7 Da isolation width, fragmented using a normalized 32% HCD of ion routing multipole and analyzed using ion trap. The top 10 MS2 fragment ions in a subsequent scan were isolated and fragmented using HCD at a 65% normalized collision energy and analyzed using an Orbitrap mass analyser at a 50,000 resolution, in the scan range of 100–500 m/z.

The proteomics raw data were searched using SEQUEST HT search engines with Proteome Discoverer 2.4 (Thermo Fisher Scientific). The following parameters were used for the analysis: Precursor mass tolerance 10 ppm, Fragment mass tolerance 0.1, Enzyme: trypsin, Mis-cleavage: −2, Fixed modification: carbamidomethylation of cysteine residues and TMT of lysine and N-terminal, Dynamic modification: oxidation of methionine. The data were filtered for 1% PSM, peptide and protein level FDR. Only unique peptides were selected for the quantification. Phospho-peptide-enriched fractions from each replicate were searched against the Uniprot protein database using the SEQUEST HT search engines with Proteome Discoverer 2.4 (Thermo Fisher Scientific). A 10-plex TMT reporter ion workflow was loaded and the following search parameters were used: trypsin protease was selected; two missed cleavages were allowed; oxidation of Met and phosphorylation of Ser/Thr/Tyr were set as variable modifications; and carbamidomethylation of Cys, TMT of lysine and N-terminal were set as fixed modifications. The mass error tolerance for MS1 and MS2 (10 ppm and 0.02 Da) was used. The data were filtered for 1% PSM, peptide and protein level FDR. For identification of the phospho-site probability, the ptmRS node was used. For proteomic data: The SEQUEST output protein group text files were processed using the Perseus software suite. The reporter ion intensities were log2 transformed and the data was normalized by the median for each sample independently. Student-T test was performed and permutation-based false discovery rate of 5% was applied to identify the differentially enriched and significant protein groups. For cluster analysis, the multiple T-test ANOVA was carried out with Benjamin Hochberg correction false discovery rate (FDR) of 5%. Significant changes were classified by *p* < 0.05 and a fold change greater than either 1.5-fold for phospho-proteomic analysis or 2-fold for total proteomic analysis.

#### Synthesis of bromoTAG-dTAG proximity-inducing chimera (BDPIC)

The full methodology for the synthesis of BDPIC is included in the Supplementary Materials (Methods S1). dTAG^V^-1 NEG,^46^ *cis*-AGB1,^45^ HDPIC (Brewer et al., joint submission) and PhosTAC7^27^ were synthesized by Natalia Shpiro as described previously.

### Quantification and statistical analysis

Statistical significance was determined using unpaired Student’s t test for 2 group comparisons and one-way ANOVA with Kruskal-Wallis’s multiple comparison test for comparing the means of >2 groups to the control. The statistical significance is denoted on graphs.

## Supplemental information

Document S1. Figures S1–S10 and Method S1
