# Supplementary material for: Targeted dephosphorylation of TFEB promotes its nuclear translocation
Source: iScience. 2024 Jun 29;27(8):110432. doi: 10.1016/j.isci.2024.110432 (PMC11284556; doi:10.1016/j.isci.2024.110432)
Supplement: Document S1. Figures S1–S10 and Method S1 [file mmc1.pdf]

iScience, Volume *27*

Supplemental information

Targeted dephosphorylation of TFEB promotes its nuclear translocation

Jin-Feng Zhao, Natalia Shpiro, Gajanan Sathe, Abigail Brewer, Thomas J. Macartney, Nicola T. Wood, Florentina Negoita, Kei Sakamoto, and Gopal P. Sapkota

Supplementary Materials

Figure S1

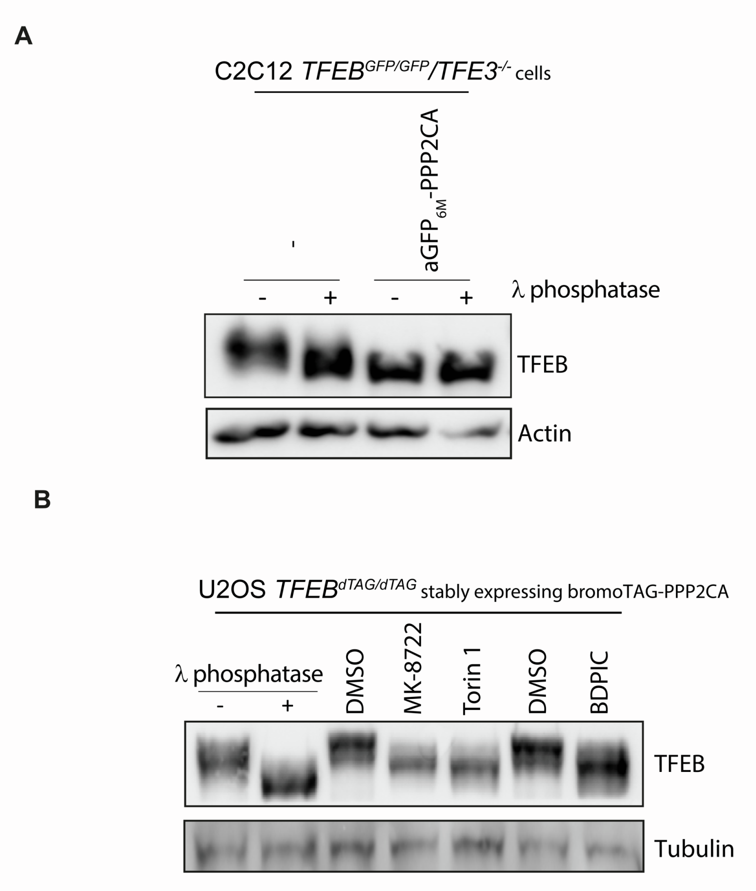

Figure S1. Comparing TFEB electrophoretic mobility to levels of phosphorylation, Related to Figure 1.

(**A**) Lysates from *TFEB*$^{GFP/GFP}$/*TFE3*$^{-/-}$ C2C12 myoblast cells were treated with or without λ-phosphatase for 30 min at 30ºC. Samples were resolved by SDS-PAGE and immunoblotted with the indicated antibodies.

(**B**) Lysates from *TFEB*$^{dTAG/dTAG}$ U2OS cells stably expressing HA-bromoTAG-PPP2CA were treated with or without λ-phosphatase for 30 min at 30ºC. Samples were resolved by SDS-PAGE and immunoblotted with the indicated antibodies.

Figure S2

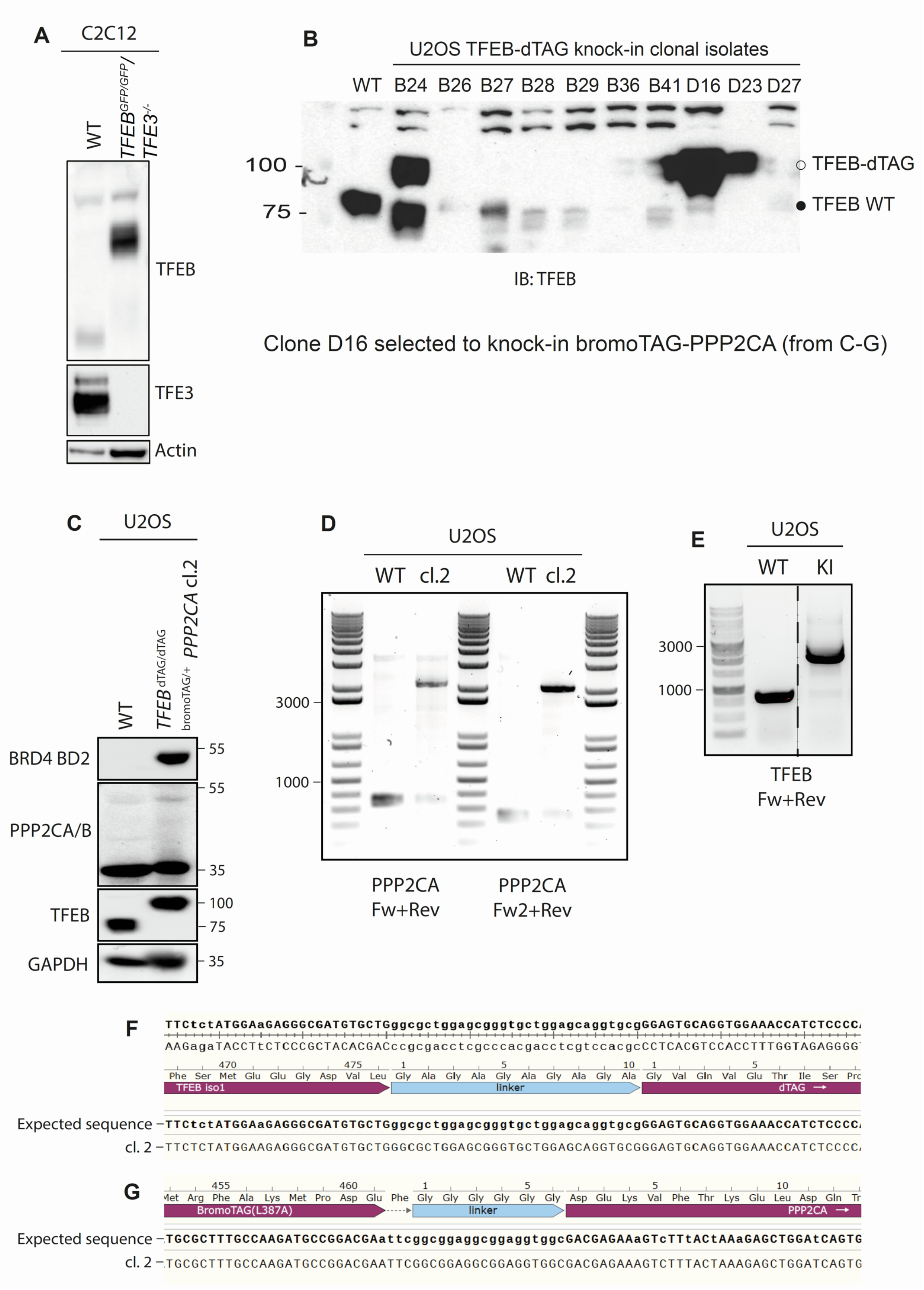

Figure S2: Validation of CRISPR/Cas9 edited cell lines used in this study, Related to Figure 1. (**A**) *TFEB*$^{GFP/GFP}$/*TFE3*$^{-/-}$ C2C12 myoblast cells were generated using a two-step CRISPR/Cas9 genome editing approaches. In the first, GFP was knocked in at the C-terminus of the *TFEB* locus homozygously and a single clone (clone 2-28) selected. In this clone, TFE3 was knocked out by CRISPR/Cas9 genome editing and a single clone (clone 1-3) isolated. The validation by immunoblotting is included. For

immunoblotting, cells were lysed before extracts (25 μg protein) were resolved by SDS-PAGE, transferred to nitrocellulose membrane and subjected to immunoblotting with the indicated antibodies.

(**B**) Immunoblotting validation of several potential *TFEB*$^{dTAG/dTAG}$ knock-in U2OS clonal isolates compared with wild-type (WT) U2OS cells. Clone D16 was selected for further experiments, and for knocking in bromoTAG-PPP2CA and subsequent validation by PCR and sequencing as detailed below.

(**C**) Immunoblotting validation of U2OS *TFEB*$^{dTAG/dTAG}$/$^{bromoTAG/+}$*PPP2CA* (clone 2) double knockin clonal isolate compared with wild-type (WT) U2OS cells.

(**D**) Confirmation of *TFEB*$^{dTAG/dTAG/bromoTAG}$/$^{+}$*PPP2CA* knock-in (clone 2) by PCR amplification of the target PPP2CA genomic region with two different sets of primers. DNA from WT U2OS cells was included as a negative control.

(**E**) Confirmation of *TFEB*$^{dTAG/dTAG}$/$^{bromoTAG/+}$*PPP2CA* knock-in by PCR amplification of the target TFEB genomic region as in (C) using the indicated primers. Samples were run on the same gel but non-relevant samples (from other clonal isolates) have been omitted from the cropped image shown.

(**F**) Confirmation of insertion of *TFEB*$^{dTAG/dTAG}$ knock-in at desired genomic locus by sequencing of the target locus in U2OS *TFEB*$^{dTAG/dTAG/bromoTAG/+}$*PPP2CA* (clone 2) cells.

(**G**) Confirmation of insertion of $^{bromoTAG/+}$*PPP2CA* knock-in at desired genomic locus by sequencing of the target locus in U2OS *TFEB*$^{dTAG/dTAG}$/$^{bromoTAG/+}$*PPP2CA* (clone 2) cells.

Figure S3

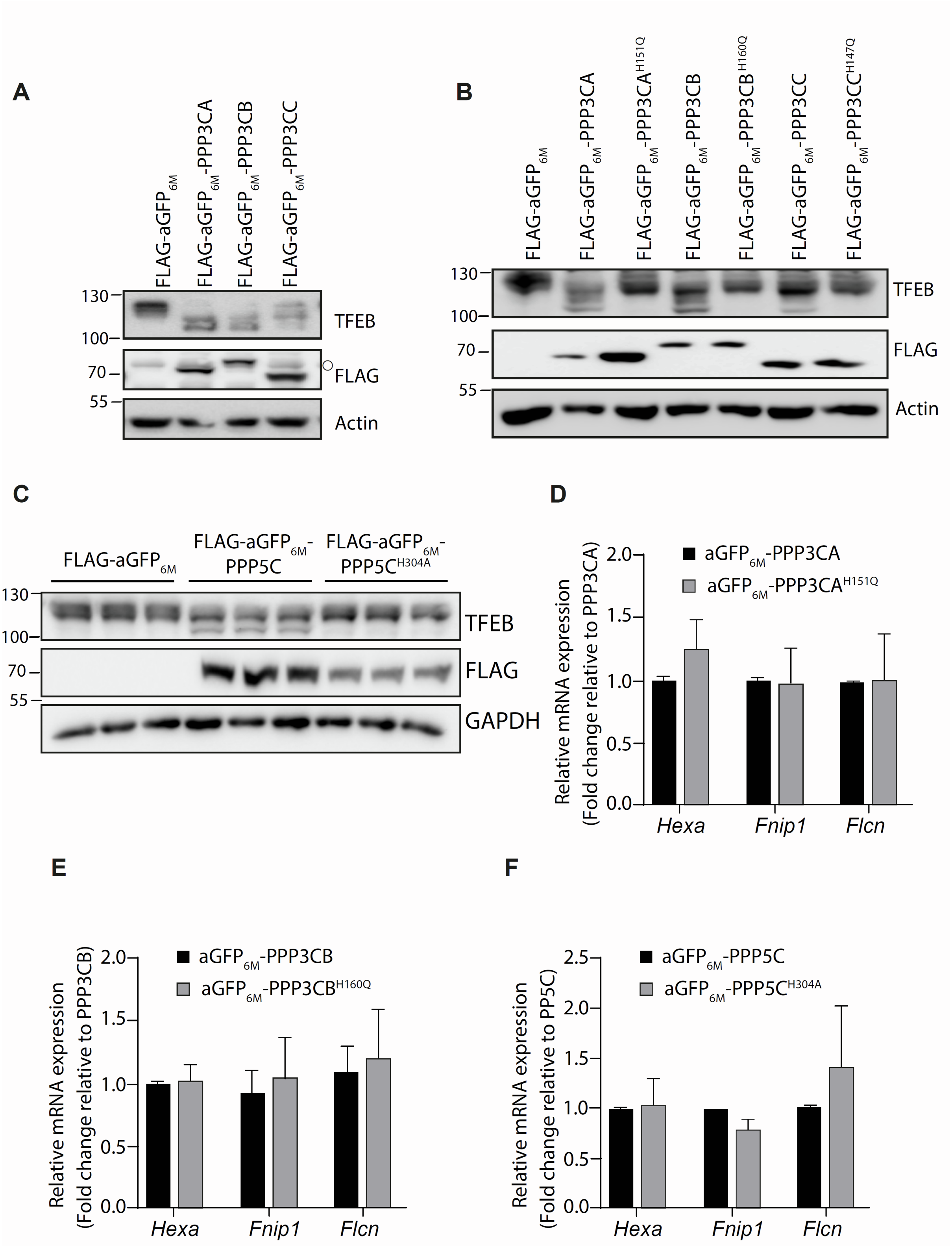

Figure S3: Different AdPhosphatases target dephosphorylation of TFEB-GFP, Related to Figure 1.

(**A**) *TFEB*$^{GFP/GFP}$ U2OS cells stably expressing FLAG-aGFP$_{6M}$-empty, FLAG-aGFP$_{6M}$-PPP3CA, FLAG-aGFP$_{6M}$-PPP3CB or FLAG-aGFP$_{6M}$-PPP3CC were lysed and extracts (25 µg protein) subjected to immunoblot analysis as indicated. ○, non-specific signal recognised by anti-FLAG antibody.

(**B**) *TFEB*$^{GFP/GFP}$ U2OS cells stably expressing FLAG-aGFP$_{6M}$, FLAG-aGFP$_{6M}$-PPP3CA or catalytic dead mutant (PPP3CA^{H151Q}), FLAG-aGFP$_{6M}$-PPP3CB or catalytic dead mutant (PPP3CB^{H160Q}) or FLAG-aGFP$_{6M}$-PPP3CC or catalytic dead mutant (PPP3CC^{H147Q}) were lysed and extracts (25 µg protein) subjected to immunoblot analysis as indicated.
(**C**) *TFEB*$^{GFP/GFP}$ U2OS cells stably expressing FLAG-aGFP$_{6M}$, FLAG-aGFP$_{6M}$-PPP5C or catalytic dead mutant (PPP5C^{H304A}) were lysed and extracts (25 µg protein) subjected to immunoblot analysis as indicated.
(**D-E**) The expression of *Hexa*, *Flcn* and *Fnip1* transcripts was measured by RT-qPCR in *TFEB*$^{GFP/GFP}$ U2OS cells stably expressing FLAG-aGFP$_{6M}$-PPP3CA or catalytic dead mutant (PPP3CA^{H151Q}) (**D**), or FLAG-aGFP$_{6M}$-PPP3CB or catalytic dead mutant (PPP3CB^{H160Q}) (**E**).
(**F**) The expression of *Hexa*, *Flcn* and *Fnip1* transcripts was measured by RT-qPCR in *TFEB*$^{GFP/GFP}$ U2OS cells stably expressing FLAG-aGFP$_{6M}$-PPP5C WT or catalytic dead mutant (PPP5C^{H304A})
All data are (A-F) representative of 3 independent experiments. Statistical analysis involved t-test with Mann-Whitney test.

Figure S4

A

Position	Phosphopeptide	Phosphosite found in PPP2CA^{H118Q} but not PPP2CA WT IPs
103-125	FAAHVSPAQG**<u>S</u>**PKPAPAASPGVR	S113 (S114 in Human)
126-165	AGHVLS**<u>T</u>**SAGNSAPN**<u>S</u>**PMAMLHISSNPEKEFDDVIDNIMR	T132 and S141 (S142 in Human)
324-346	VHGLP**<u>TTS</u>**PSGVNMAELAQQVVK(T/S)	T329, T330 or S331 (T330, T331 or S332 in Human)
464-475	RS**<u>S</u>**F**<u>S</u>**MEEGDVL	S466 and S468 (S467 and S469 in Human)

B

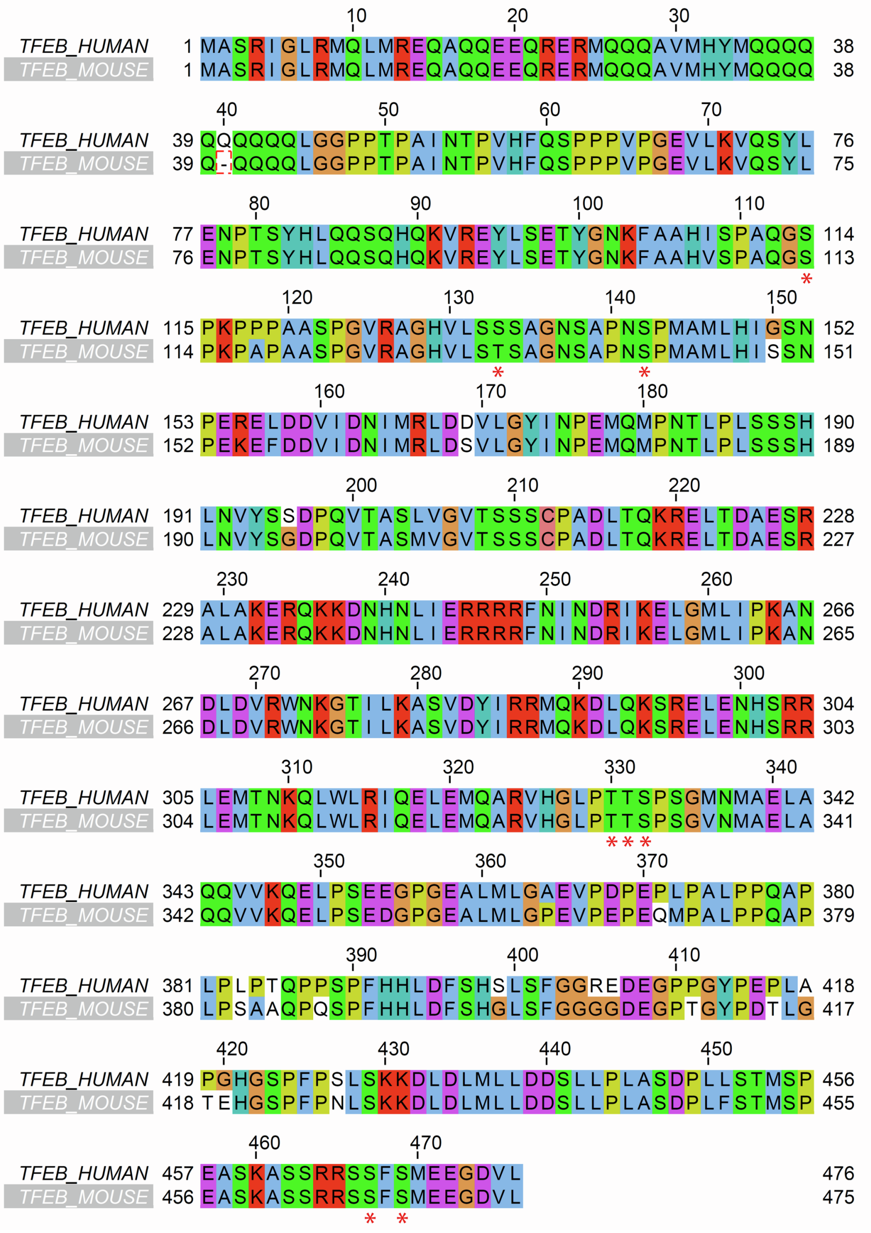

Figure S4: Phospho-peptides on TFEB-GFP identified by MS in FLAG-aGFP$_{6M}$-PPP2CA^{H118Q} IPs that were absent from FLAG-aGFP$_{6M}$-PPP2CA IPs, Related to Figure 1.

(**A**) *TFEB$^{GFP/GFP}$/TFE3$^{-/-}$* C2C12 cells stably expressing FLAG-aGFP$_{6M}$-PPP2CA or FLAG-aGFP$_{6M}$-PPP2CA^{H118Q} were lysed, and extracts (5 mg protein) subjected to anti-FLAG IP. TFEB-GFP associated with the FLAG-aGFP$_{6M}$-AdPhosphatase IPs was analysed by tandem mass spectrometry (MS/MS) for phospho-peptide analysis. The identified phospho-peptides on TFEB are indicated.

(**B**) Sequence alignment of the human and mouse TFEB proteins. The alignment was performed using Jalview. The asterisks represent identified phosphorylation sites in mouse TFEB sequence.

Figure S5

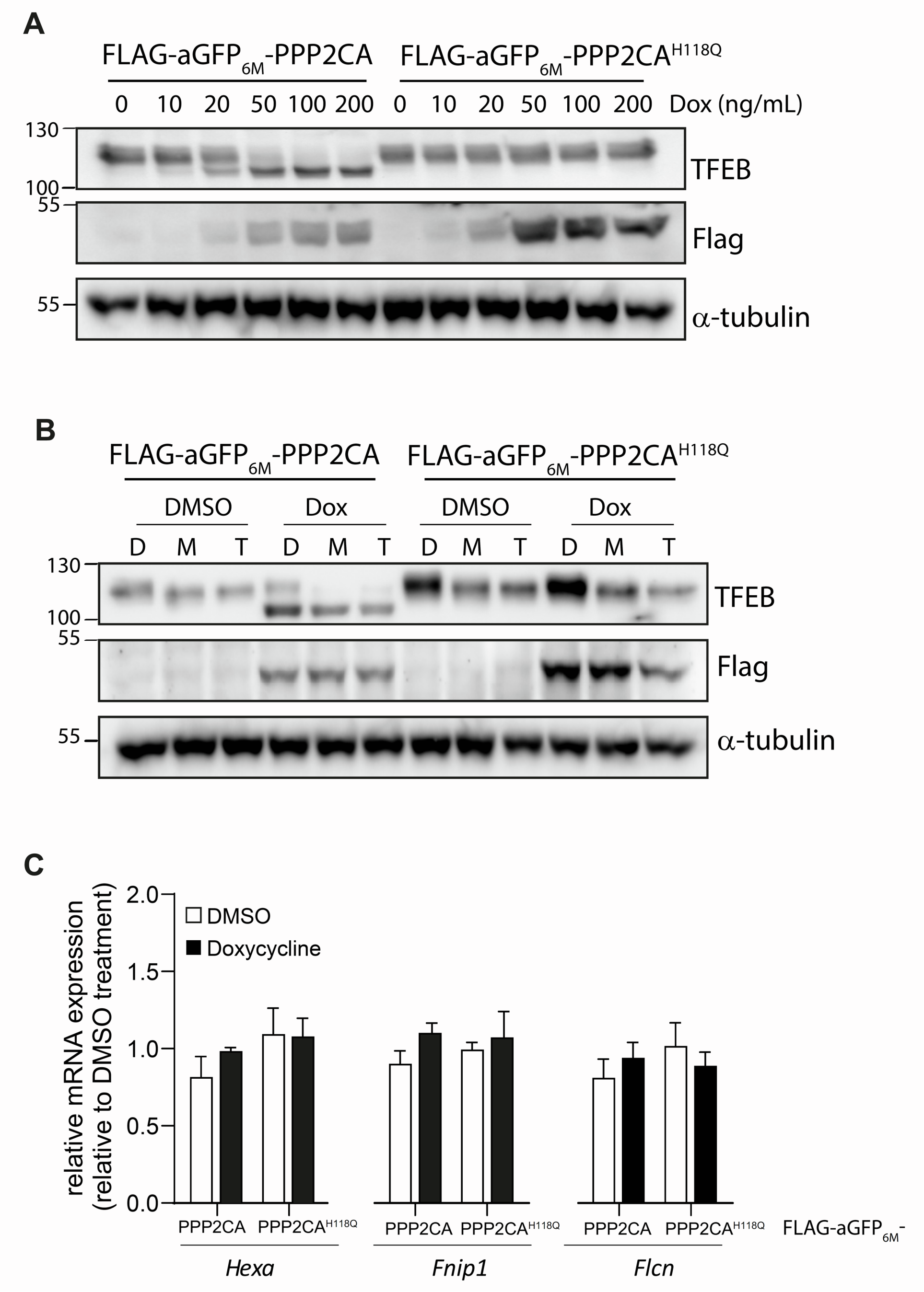

Figure S5: Inducible AdPhosphatase system for tractable TFEB-GFP dephosphorylation, Related to Figure 1.

(**A-B**) *TFEB$^{GFP/GFP}$/TFE3$^{-/-}$* C2C12 cells were retrovirally transduced with FLAG-aGFP$_{6M}$-PPP2CA or FLAG-aGFP$_{6M}$-PPP2CA^{H118Q} using the tetracycline-inducible (Tet-One system) vectors. (**A**) Cells were treated with increasing concentrations of doxycycline (Dox) for 24 h prior to lysis. Immunoblot analysis on extracts (25 µg protein) was performed with the indicated antibodies. (**B**) Cells were treated with 100 ng/ml doxycycline for 24 h. Cells were cultured with DMSO (D), 10 µM MK-8722 (M) or 100 nM Torin 1 (T) for further 4 h before lysis. Immunoblot analysis on extracts (25 µg protein) was performed with the indicated antibodies. (**A-B**) Data representative of 3 independent experiments. (**C**) Cells were treated with 100 ng/ml doxycycline (Dox) for 24 h prior to lysis. The expression of *Hexa*, *Fnip1*, and *Flcn* transcripts was examined by RT-qPCR. Data are mean ± SEM from 3 independent experiments. Statistical analysis involved t-test with Mann-Whitney test.

Figure S6

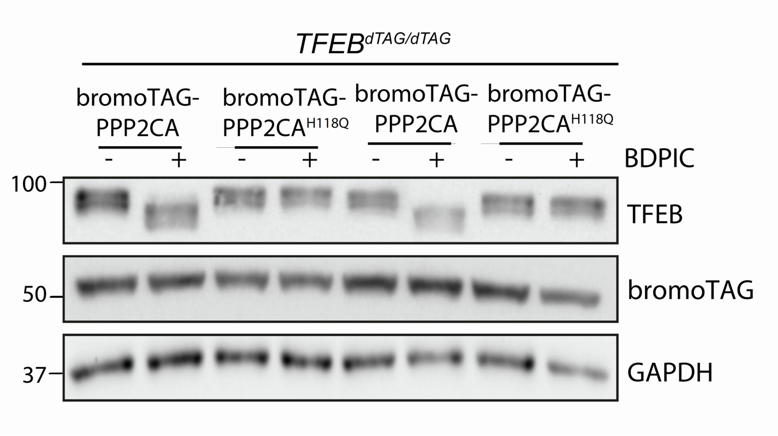

Figure S6. Targeting TFEB-dTAG dephosphorylation via BDPIC-mediated proximity with bromoTAG-PPP2CA or bromoTAG-PPP2CA^{H118Q} expressed at the same levels Related to Figure 2.

TFEB$^{dTAG/dTAG}$ U2OS cells were transduced with lower titre of retroviruses encoding the expression of HA-bromoTAG-PPP2CA (1:16 dilution) or undiluted HA-bromoTAG-PPP2CA^{H118Q} encoding retroviral media for 48 h. After puromycin selection, stable lines were treated with DMSO or BDPIC (100 nM) for 2 h before lysis. Immunoblot analysis on extracts (25 µg protein) was performed with the indicated antibodies.

Figure S7

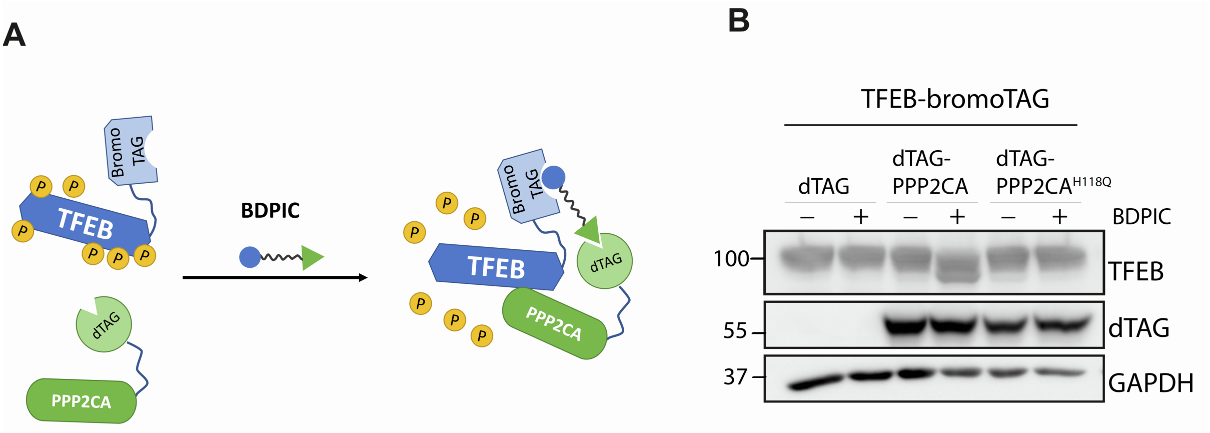

Figure S7. Targeting TFEB-bromoTAG dephosphorylation via BDPIC-mediated proximity with dTAG-PPP2CA, Related to Figure 2.
(**A**) Schematic representation of BDPIC-mediated induction of proximity between TFEB-bromoTAG and dTAG-PPP2CA.
(**B**) U2OS cells stably co-expressing TFEB-bromoTAG and dTAG, dTAG-PPP2CA or dTAG-PPP2CA^{H118Q} were treated with BDPIC for 24 h before lysis. Immunoblot analysis was performed on extracts (10 µg protein) with the indicated antibodies. Data representative of 3 independent experiments.

Figure S8

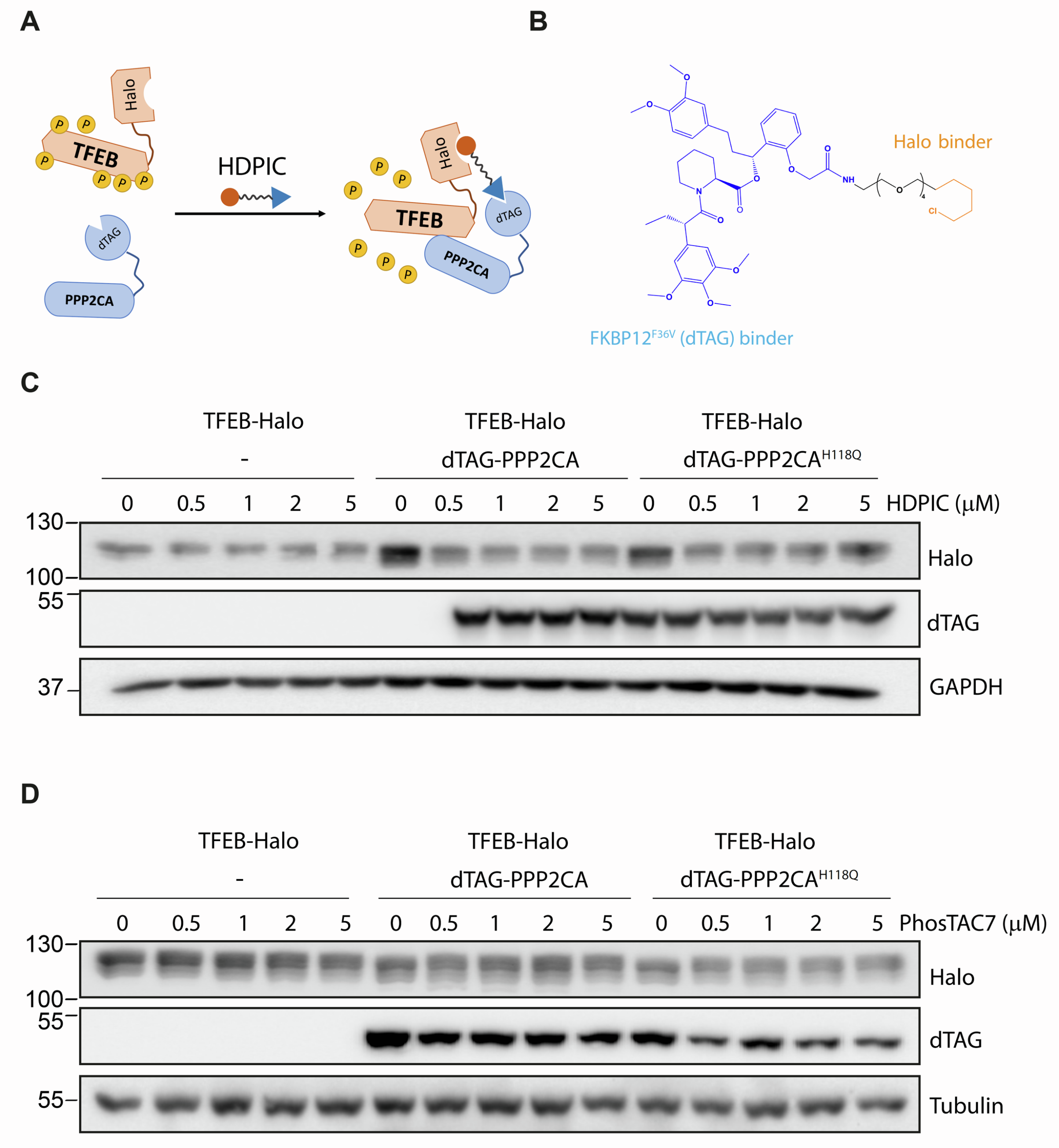

Figure S8. Effect of Halo-tag dTAG proximity-inducing chimera (HDPIC) and PhosTAC7 on targeted dephosphorylation of TFEB-Halo via recruitment of dTAG-PPP2CA, Related to Figure 2.

(**A**) Schematic representation of HDPIC-mediated induction of proximity between TFEB-Halo and dTAG-PPP2CA.

(**B**) Structures of HDPIC with FKBP12^{F36V} (dTAG and Halo-tag binding warheads connected with the indicated PEG linker.

(**C-D**) U2OS cells stably expressing TFEB-Halo or those co-expressing TFEB-Halo and dTAG-PPP2CA or dTAG-PPP2CA^{H118Q} were generated. Cells were treated with HDPIC (**C**) or PhosTAC7 (**D**) at indicated concentrations for 24 h prior to lysis. Immunoblotting analysis was performed on extracts (10 μg protein) with the indicated antibodies. All data representative of 3 independent experiments.

Figure S9

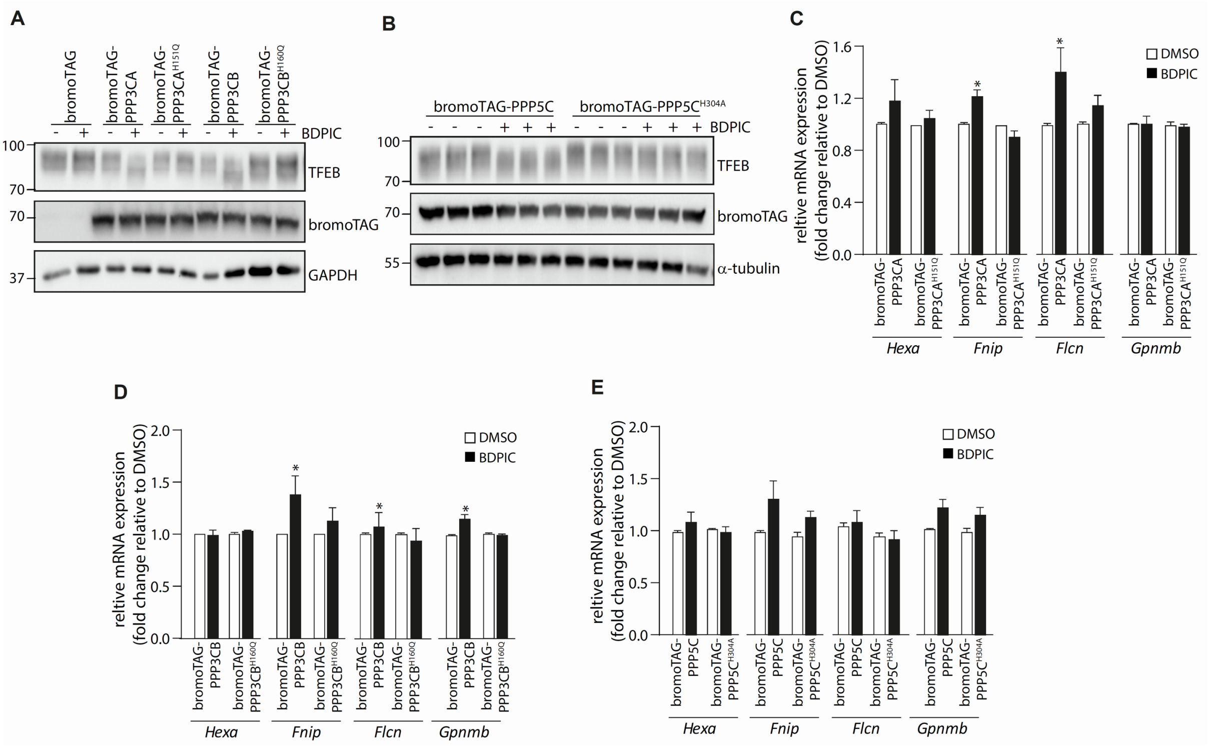

Figure S9. Targeting TFEB-dTAG dephosphorylation via BDPIC-mediated proximity with bromoTAG-phosphatases, Related to Figure 4.

(**A**) $TFEB^{dTAG/dTAG}$ U2OS cells stably expressing HA-bromoTAG-PPP3CA or catalytic dead mutant (PPP3CA^{H151Q}), and HA-bromoTAG-PPP3CB or catalytic dead mutant (PPP3CB^{H160Q}) were treated with DMSO or BDPIC (100 nM, 2 h) prior to lysis. Immunoblotting analysis was performed on extracts (25 µg protein) with the indicated antibodies.

(**B**) $TFEB^{dTAG/dTAG}$ U2OS cells stably expressing HA-bromoTAG-PPP5C or catalytic dead mutant (PPP5C^{H304A}), were treated with DMSO or BDPIC (100 nM, 2 h) prior to lysis. Immunoblotting analysis was performed on extracts (25 µg protein) with the indicated antibodies.

(**C**) The expression of *Hexa*, *Flcn, Fnip1 and Gpnmb* transcripts was measured by RT-qPCR in $TFEB^{dTAG/dTAG}$ U2OS cells stably expressing HA-bromoTAG-PPP3CA or catalytic dead mutant (PPP3CA^{H151Q}).

(**D**) The expression of *Hexa*, *Flcn, Fnip1 and Gpnmb* transcripts was measured by RT-qPCR in $TFEB^{dTAG/dTAG}$ U2OS cells stably expressing HA-bromoTAG-PPP3CB or catalytic dead mutant (PPP3CB^{H160Q}).

(**E**) The expression of *Hexa*, *Flcn, Fnip1 and Gpnmb* transcripts was measured by RT-qPCR in $TFEB^{dTAG/dTAG}$ U2OS cells stably expressing HA-bromoTAG-PPP5C or catalytic dead mutant (PPP5C^{H304A}).

All data are (A-E) representative of 3 independent experiments. All quantitative data are mean ± SEM from 3 independent experiments. * $P < 0.05$ compared with DMSO treatment. Statistical analysis involved t-test with Mann-Whitney test.

Figure S10

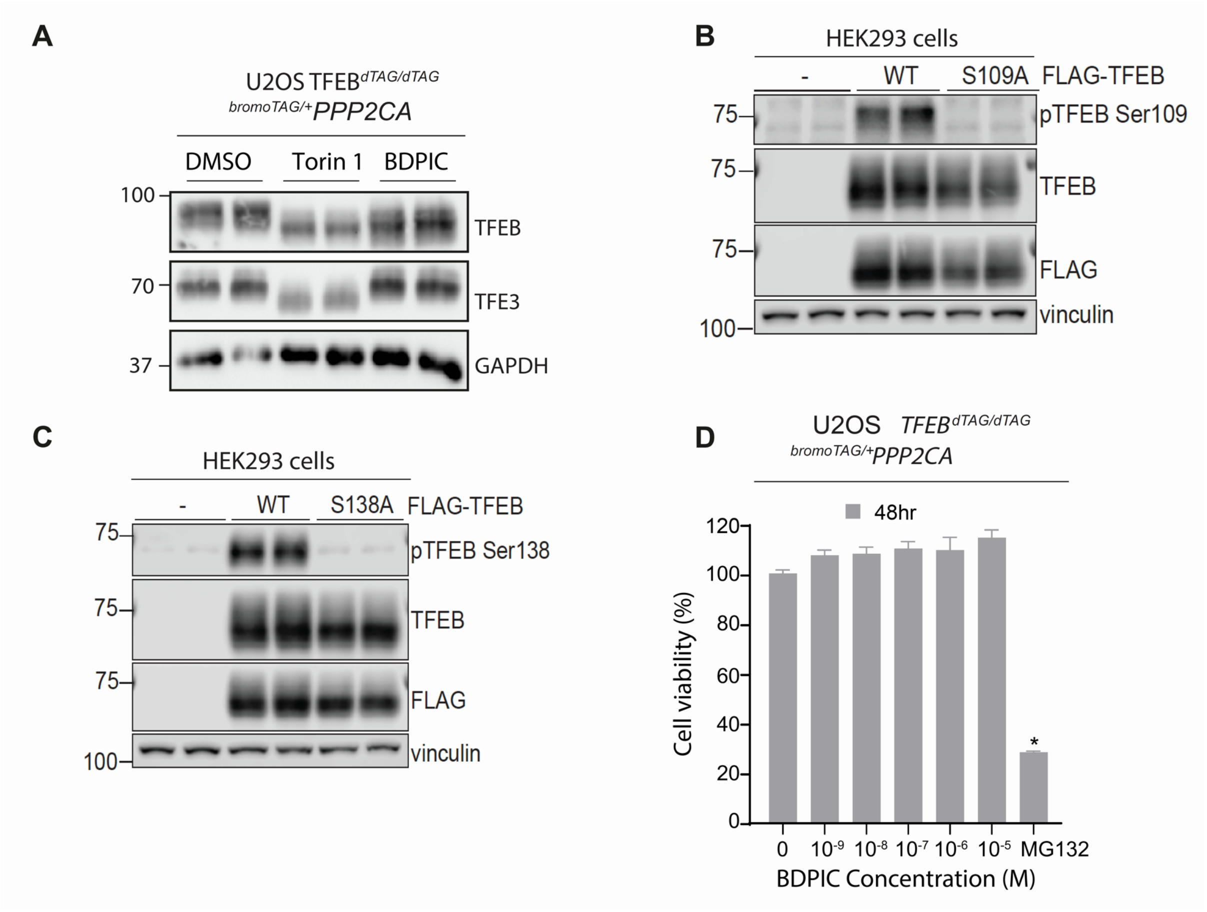

Figure S10: Specificity and toxicity of BDPIC in cells and assessing the on-target engagement of newly generated phospho-TFEB antibodies, Related to Figure 5.

(**A**) U2OS *TFEB*$^{dTAG/dTAG/bromoTAG/+}$*PPP2CA* (clone 2) cells were treated for 2 h with BDPIC (100 nM), Torin 1 (100 nM) or DMSO before cells were lysed and extracts (20 µg protein) were resolved by SDS-PAGE, transferred to nitrocellulose membrane and subjected to immunoblotting with the indicated antibodies.

(**B-C**) HEK293 cells were transiently transfected with plasmids encoding TFEB-FLAG wild type (WT), Ser109Ala (**B**) or Ser138Ala (**C**) mutants. Protein lysates were collected 48 h post-transfection and subjected to immunoblot analysis of TFEB-FLAG and phosphorylated TFEB-FLAG on Ser109 (**B**) or Ser138 (**C**) using the specified antibodies. Vinculin was used as a loading control.

(**D**) Cells from (**A**) were treated with DMSO or BDPIC at the indicated concentrations for 48 h. A cell viability assay was performed. Values were normalized with the DMSO group. All quantitative data are mean ± SEM from 5 independent experiments. MG132 (20 µM) treatment was used as positive control for cytotoxicity. * $P < 0.05$. Statistical analysis involved One-way ANOVA with Kruskal-Wallis test.

Methods S1: Chemistry methods, Synthesis of BDPIC, related to Figure 2, 3, 4, 5 and 6.
To ensure complete access of the full methodology for BDPIC synthesis, we include the identical detailed methodology in this manuscript as well as in the manuscript by *Brewer et al* that was jointly submitted for publication.

Abbreviations:

AcOH	Acetic acid
DCE	1,2-Dichloroethane
DCM	Dichloromethane
DIC	*N*,*N* -Diisopropylcarbodiimide
DIPEA	*N*,*N*-Diisopropylethylamine
DMAP	4-(Dimethylamino)pyridine
DMF	*N,N*-Dimethylformamide
DMSO	Dimethyl sulfoxide
Et_3N	Triethylamine
EtOAc	Ethyl acetate
Et_3SiH	Triethylsilane
LiHMDS	Lithium bis(trimethylsilyl)amide
MeCN	Acetonitrile
MeOH	Methanol
NH_4Cl	Ammonium chloride
$Pd(OAc)_2$	Palladium(II) acetate
PyBOP	Benzotriazole-1-yl-oxy-tris-pyrrolidino-phosphonium hexafluorophosphate
TFA	Trifluoroacetic acid
THF	Tetrahydrofuran
T3P	Propanephosphonic acid anhydride

General comments
All chemicals were purchased from commercial vendors and used without further purification. Flash column chromatography and Prep HPLC were performed by using Buchi PrepChrom C-700, prepacked Buchi Sepacore Flash Cartridges and HPLC C18 column Gemini NY(RP)C18 110, 21.2×150 mm, 10 μn particle size. Details about the conditions for preparative HPLC are provided in the experimental procedures. NMR spectra were recorded on a Bruker Ascend 500 MHz. Chemical shifts are reported in parts per million referenced to residual solvent peaks ($CDCl_3$= 7.26 ppm). Only the chemical shifts of the major rotamer are reported. The following abbreviations were used in reporting spectra, s (singlet), d (doublet), t (triplet), q (quartet), m (multiplet), dd (doublet of doublets), bs (broad signal). Low resolution mass spectra and analytical HPLC traces were recorded on an Agilent Technologies 1200 series HPLC connected to an Agilent Technologies 6130 quadrupole LC/MS, connected to an Agilent diode array detector. The column used was a Waters XBridge column (50 mm × 2.1 mm, 3.5 μm particle size), with a gradient from 5 % to 95% of acetonitrile in water (with 0.1 % of formic acid or aqueous ammonia solution) over 3 or 7 minutes. The flow rate was 0.7 mL/min.

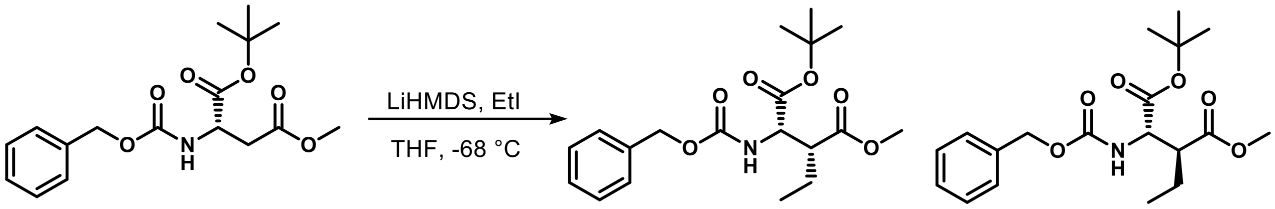

1 2 3

1-(tert-butyl) 4-methyl (2S,3R)-2-(((benzyloxy)carbonyl)amino)-3-ethylsuccinate (**2**) and 1-(tert-butyl) 4-methyl (2S,3S)-2-(((benzyloxy)carbonyl)amino)-3-ethylsuccinate (**3**)
To a solution of 1-(tert-butyl) 4-methyl ((benzyloxy)carbonyl)-L-aspartate **1**[1] (9.38 g, 27.80 mmol) in 250 mL of anhydrous THF was added dropwise 1.3 M solution of LiHMDS in THF (45 mL, 58.5 mmol) at -68°C.The reaction mixture was stirred at -68°C for 1h, then at -40°C for 1h, and cooled down again to -68°C. Iodoethane (5.6 mL, 10.86 g, 69.62 mmol) was then added dropwise and the reaction was stirred for 1h at -10°C, quenched with saturated NH_4Cl solution (75 mL) and extracted with EtOAc (2 x 100 mL). The organic layers were washed with brine (75 mL), combined, and concentrated. The crude residue (10 g) was purified by flash column chromatography on silica (200 g), gradient from petroleum

spirit to 40% EtOAc in petroleum spirit to afford compound **2** (5.79 g, 15.84 mmol, 57%) and **3** (2.28g, 6.23 mmol, 22%).
2:^{1}H NMR (500 MHz, $CDCl_3$,): δ = 7.40-7.37 (m, 4H); 7.36-7.30 (m, 1H); 5.70 (d, J=9.3Hz, 1H); 5.15 (s, 2H); 4.55 (dd, J=4.0Hz, J=9.75Hz, 1H); 3.69 (s, 3H); 2.96 (ddd, J=4.0Hz, 1H); 1.85-1.73 (m, 1H); 1.64-1.55 (m, 1H); 1.45 (s, 9H); 1.01 (t, J=7.4Hz, 3H).
3: ^{1}H NMR (500 MHz, $CDCl_3$,): δ = 7.40-7.37 (m, 4H); 7.36-7.31 (m, 1H); 5.49 (d, J=7.2Hz, 1H); 5.12 (s, 2H); 4.56 (dd, J=5.0Hz, J=8.3Hz, 1H); 3.71 (s, 3H); 2.78-2.72 (m, 1H); 1.90-1.80 (m, 1H); 1.62-1.53 (m, 1H); 1.48 (s, 9H); 0.98 (t, J=7.2Hz, 3H).
$C_{19}H_{27}NNaO_6$, expected for $[M+Na]^+$ 388.17, found $[M+Na]^+$ 388.0

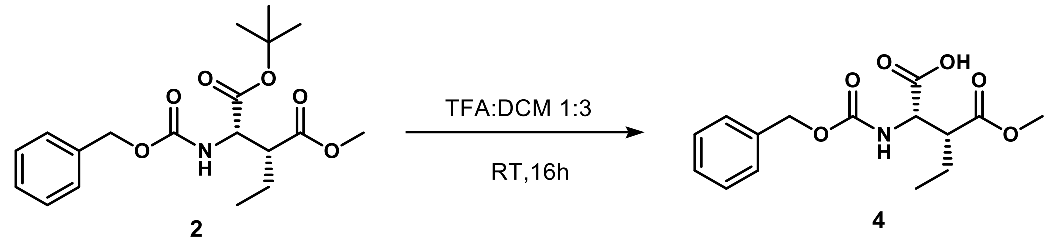

(2S,3R)-2-(((benzyloxy)carbonyl)amino)-3-(methoxycarbonyl)pentanoic acid (**4**)
To a solution of 1-(tert-butyl) 4-methyl (2S,3R)-2-(((benzyloxy)carbonyl)amino)-3-ethylsuccinate (**2**) (2.10 g, 5.75 mmol) in DCM (5 mL) was added solution of TFA in DCM (40 mL, 1:3 v/v) and the reaction mixture was stirred at RT for 16h and concentrated. Crude residue was purified by RP C18 flash column chromatography (SVP D40-RP18 25-40 μm 90 g), in a gradient of $MeOH/H_2O$ (5% to 95%) to afford compound **4** (1.39 g, 4.50 mmol, 78%).
^{1}H NMR (500 MHz, $CDCl_3$,): δ = 7.40-7.33 (m, 5H); 5.89 (d, J=9.4Hz, 1H); 5.17 (s, 2H); 4.68 (dd, J=3.6Hz, J=9.4Hz, 1H); 3.73 (s, 3H); 3.08 (ddd, J=3.5Hz, 1H); 1.89-1.80 (m, 1H); 1.70-1.60 (m, 1H); 1.05 (t, J=7.4Hz, 3H).
$C_{15}H_{19}NNaO_6$, expected for $[M+Na]^+$ 332.11, found $[M+Na]^+$ 332.1

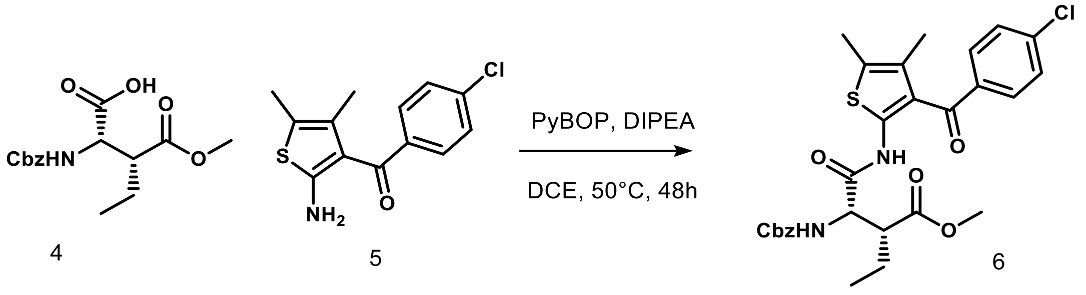

Methyl (2R,3S)-3-(((benzyloxy)carbonyl)amino)-4-((3-(4-chlorobenzoyl)-4,5-dimethylthiophen-2-yl)amino)-2-ethyl-4-oxobutanoate (**6**).
To a solution of **4** (1.53 g, 4.94 mmol) in DCE (45 mL) were added **5** [2] (1.31 g, 4.94 mmol), DIPEA (4.30 mL, 24.7 mmol) and PyBOP (6.42 g, 12.35 mmol) at RT. The reaction mixture was stirred at 50°C for 48 h, cooled to RT, diluted with water (25 mL) and extracted with DCM (2×50 mL). The organic layer was washed with brine (25 mL) and concentrated. The crude residue was purified by column chromatography on silica (160 g), gradient from petroleum spirit to 40% EtOAc in petroleum spirit to give crude material (1.57 g) which was further purified by RP C18 flash column chromatography (SVP D40-RP18 25-40 μm 90 g) in a gradient of $MeCN/H_2O$ (5% to 95%) to afford compound **6** (1.13 g, 2.02 mmol, 41%).
^{1}H NMR (500 MHz, $CDCl_3$,): δ = 11.74 (s,1H); 7.56(d, J=8.4Hz, 2H); 7.45 (d, J=8.4Hz,4H); 7.34-7.29 (m, 3H); 6.24(d, J=9.4Hz,1H); 5.30 (d, J=12.1Hz, 1H); 5.18 (d, J=12.1Hz,1H); 4.75 (dd, J=3.4Hz, J=9.6Hz, 1H); 3.68 (s, 3H); 3.34-3.27 (m, 1H); 2.27 (s, 3H); 1.89-1.79 (m, 1H); 1.71 (s, 3H); 1.67-1.59 (m, 1H); 1.05 (t, J=7.4Hz, 3H).
$C_{28}H_{29}ClN_2NaO_6S$, expected for $[M+Na]^+$ 579.13, found $[M+Na]^+$ 579.0

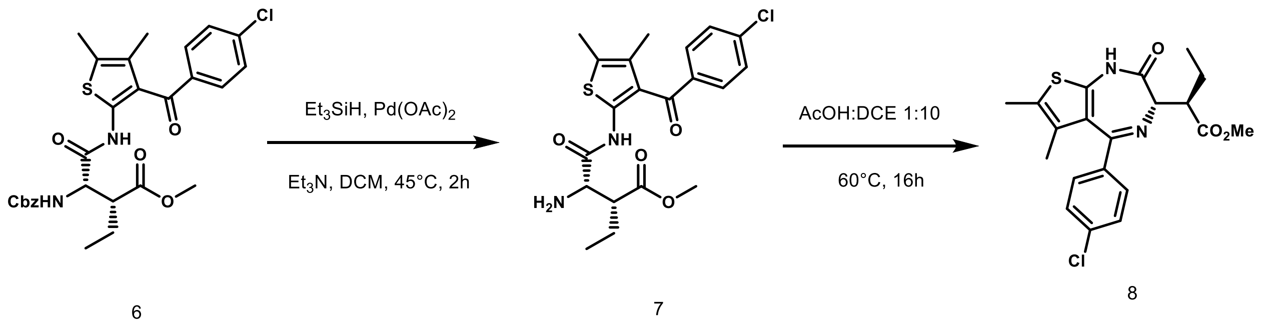

Methyl (R)-2-((S)-5-(4-chlorophenyl)-6,7-dimethyl-2-oxo-2,3-dihydro-1H-thieno[2,3-e][1,4]diazepin-3-yl)butanoate (**8**)
To a solution of **6** (0.98 g, 1.76 mmol) in DCM (18 mL) were added $Pd(OAc)_2$ (0.04 g, 0.17 mmol) and Et_3N (0.1 mL, 0.71 mmol) and the reaction was heated at 45°C for 5 min while triethylsilane (1.40 mL,1.01 g, 8.76 mmol) was added dropwise. The reaction mixture was further stirred at 45°C for 2 h, cooled to RT, diluted with DCM (20 mL), filtered through Celite, and concentrated. The crude residue of methyl(2R,3S)-3-amino-4-((3-(4-chlorobenzoyl)-4,5-dimethylthiophen-2-yl)amino)-2-ethyl-4-oxobutanoate (**7**) was used in the next step without further purification.
$C_{20}H_{24}ClN_2O_4S$, expected for $[M+H]^+$ 423.11, found $[M+H]^+$ 423.1.
To a solution of **7** (crude residue 1.1 g) in DCE (10 mL) 10% of acetic acid was added and the reaction mixture was kept at 60°C for 16h and concentrated. The crude residue was purified by flash column chromatography on silica (160 g), gradient from petroleum spirit to 50% EtOAc in petroleum spirit to afford compound **8** (0.37g, 0.91 mmol, 51% for two steps) as yellow shiny crystals. Enantiomeric purity of **8** was determined as 99% *ee* on chiral column CHIRALPAC IA (20x250 mm,5 µn particle size, DAICEL), 20% EtOAc in heptane, 220 nm, v=6 mL/min with t_R=15.1 min.
^{1}H NMR (500 MHz, $CDCl_3$,): δ = 8.70 (br s,1H); 7.34 (m, 4H); 3.88 (d, J=10.4Hz, 1H); 3.83 (s, 3H); 3.66 (ddd, J=4.5Hz, 1H); 2.31 (s, 3H); 1.97-1.89 (m, 1H); 1.62 (s, 3H); 1.63-1.57 (m, 1H); 1.03 (t, J=7.5Hz, 3H).
$C_{20}H_{22}ClN_2O_3S$, expected for $[M+H]^+$ 405.10, found $[M+H]^+$ 405.1
$[\alpha]^{20}_D$ = +44.1 (c 0.5, $CHCl_3$)

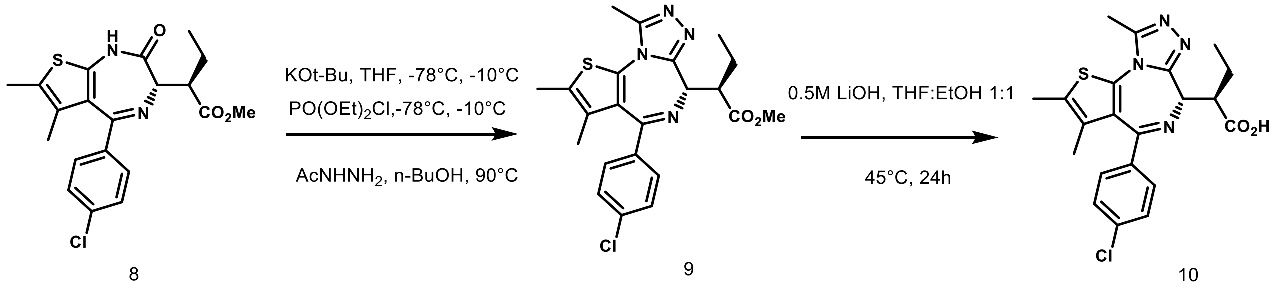

Methyl (R)-2-((S)-4-(4-chlorophenyl)-2,3,9-trimethyl-6H-thieno[3,2-f][1,2,4]triazolo[4,3-a][1,4]diazepin-6-yl)butanoate (**9**).
The title compound was obtained as described in [3] starting from **8** (0.25 g, 0.61 mmol) in 73% yield (0.20 g, 0.45mmol) as white crystals. Enantiomeric purity of **9** was determined as 99% *ee* on chiral column CHIRALPAC IA (20x250 mm,5 µn particle size, DAICEL), 20% EtOAc in heptane, 220 nm, v=6 mL/min with t_R=19.2 min.

^{1}H NMR (500 MHz, $CDCl_3$,): δ = 7.34 (m, 4H); 4.26 (d,J=10.9Hz,1H); 4.01 (ddd, J=3.8Hz, 10.7Hz, 10.9Hz, 1H); 3.87 (s, 3H); 2.69 (s, 3H); 2.44 (s, 3H); 2.25-2.15 (m, 1H); 1.70 (s, 3H); 1.74-1.63 (m, 1H); 1.04 (t, J=7.4Hz, 3H).
^{13}C NMR (126 MHz, $CDCl_3$) δ = 175.4, 163.2, 154.45,149.9, 149.8, 136.8, 136.6, 132.1, 130.9, 130.5, 129.8, 128.7, 59.4, 51.6, 49.7, 23.3, 14.5, 13.2, 11.9, 11.7
$C_{22}H_{24}ClN_4O_2S$, expected for $[M+H]^+$ 443.13, found $[M+H]^+$ 443.1
$[\alpha]^{20}_D$ = +70.1 (c 0.5, $CHCl_3$)

To a solution of **9** (0.20 g, 0.45 mmol) in a mixture of THF:EtOH 1:1 (5 mL) was added 0.5M solution of LiOH (2.7 mL, 1.35 mmol) and the mixture was stirred at 45°C for 24 h, cooled to RT, acidified with 1M

HCl (1.5 mL, 1.5 mmol), and concentrated. To a crude residue was added water (5 mL) and the precipitate was collected on sintered glass filter. It was purified by RP C18 flash column chromatography (SVP D40-RP18 25-40 µm 90 g), in a gradient of MeCN/H_2O (5% to 95%) to afford compound **10** (0.115 g, 0.26 mmol, 59%).
^{1}H NMR (500 MHz, $CDCl_3$,): δ = 7.44 (d, J=8.6Hz, 2H); 7.36(d, J-8.6Hz, 2H); 4.25 (d, J=6.1, 1H); 3.73-3.63 (m, 1H); 2.71 (s, 3H); 2.46 (s, 3H); 2.12-2.02 (m, 1H); 2.00-1.90 (m, 1H); 1.74 (s, 3H); 1.12 (t, J=7.4Hz, 3H).
^{13}C NMR (126 MHz, $CDCl_3$) δ = 174.8, 164.7, 154.8,150.0, 137.4, 135.8, 132.4, 131.4, 131.2, 130.1, 128.8, 58.0, 48.4, 23.8, 14.7, 13.2, 11.8

$C_{21}H_{22}ClN_4O_2S$, expected for $[M+H]^+$ 429.12, found $[M+H]^+$ 429.1
$[\alpha]^{20}_D$ = +103.5 (c 0.5, $CHCl_3$)

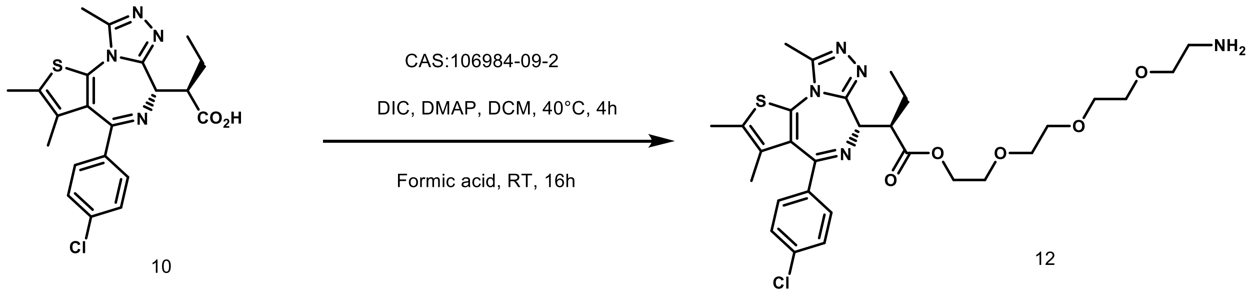

10 12

2,2-dimethyl-4-oxo-3,8,11,14-tetraoxa-5-azahexadecan-16-yl(R)-2-((S)-4-(4-chlorophenyl)-2,3,9-trimethyl-6H-thieno[3,2-f][1,2,4]triazolo[4,3-a][1,4]diazepin-6-yl)butanoate (**11**).
To a solution of **10** (0.030 g, 0 07 mmol) in DCM (2 mL) was added DIC (0.043 mL, 0.035g, 0.277 mmol), DMAP (0.011 g, 0.09 mmol) and the mixture was stirred at RT for 30 min. A solution of t-butyl-N-(2-(2-[2-(2-hydroxyethoxy)ethoxy]ethoxy)ethyl)carbamate (0.082g, 0.28 mmol) in DCM (0.5 ml) was then added and the reaction was heated at 40°C for 4 h and concentrated. The crude residue (0.15 g) was purified on HPLC RP C18 column with gradient MeCN/H_2O (5% to 95%) to give **11** (0.028 g, 0.039 mmol, 56%) as solid.
^{1}H NMR (500 MHz, $CDCl_3$,): δ = 7.36 (d, J=8.6Hz, 2H); 7.33(d, J-8.6Hz, 2H); 5.03 (br s, 1H); 4.49-4.37 (m, 2H); 4.27 (d, J=10.8Hz, 1H); 4.02 (ddd, J=3.6Hz, 1H); 3.86-3.78 (m, 2H); 3.74-3.70 (m, 2H); 3.68-3.61 (m, 6H); 3.55 (t, J=5.2Hz, 2H); 3.35-3.30 (m, 2H); 2.68 (s, 3H); 2.44 (s, 3H); 2.25-2.16 (m, 1H), 1.76-1.66 (m, 1H); 1.71 (s, 3H); 1.46 (s 9H); 1.06 (t, J=7.4Hz, 3H).
$C_{34}H_{47}ClN_5O_7S$, expected for $[M+H]^+$ 704.29, found $[M+H]^+$ 704.3

2-(2-(2-(2-aminoethoxy)ethoxy)ethoxy)ethyl(R)-2-((S)-4-(4-chlorophenyl)-2,3,9-trimethyl-6H-thieno[3,2-f][1,2,4]triazolo[4,3-a][1,4]diazepin-6-yl)butanoate (**12**)
A solution of **11** (0.028 g, 0.039 mmol) in formic acid (1 mL) was kept at RT for 16 h. The volatiles were removed in vacuo to yield **12** (0.028 g, 0.039 mmol), the crude residue was used in the next step without further purification.

$C_{29}H_{39}ClN_5O_5S$, expected for $[M+H]^+$ 604.24, found $[M+H]^+$ 604.2

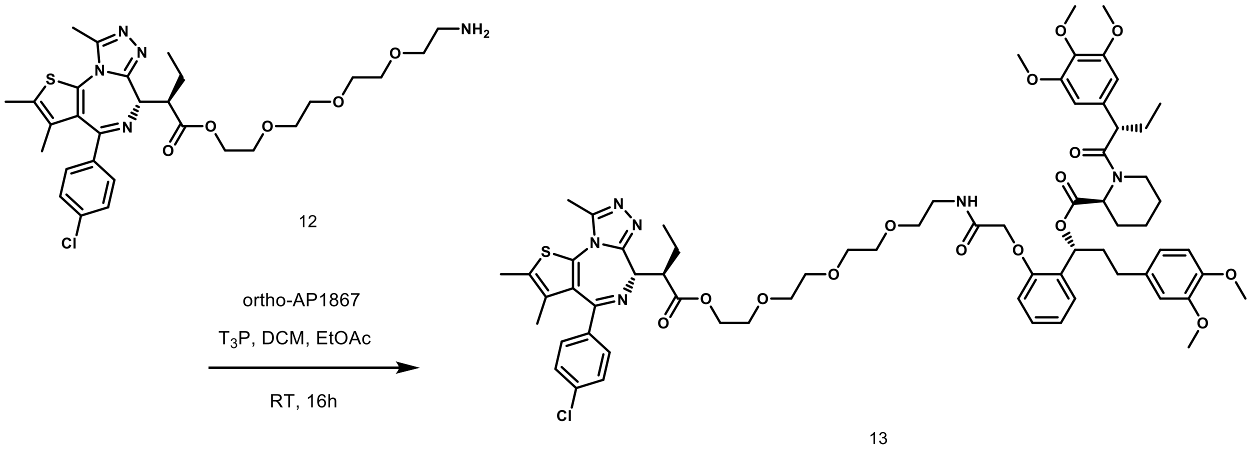

(R)-1-(2-(((R)-17-((S)-4-(4-chlorophenyl)-2,3,9-trimethyl-6H-thieno[3,2-f][1,2,4]triazolo[4,3-a][1,4]diazepin-6-yl)-2,16-dioxo-6,9,12,15-tetraoxa-3-azanonadecyl)oxy)phenyl)-3-(3,4-dimethoxyphenyl)propyl (S)-1-((S)-2-(3,4,5-trimethoxyphenyl)butanoyl)piperidine-2-carboxylate (**13**).
To a solution of acid **ortho-AP1867** [4] (0.027 g, 0.038 mmol) in DCM (0.5 mL) were added a solution of **12** (0.028g, 0.038 mmol) in DCM (1 mL), DIPEA (0.093 mL, 0.53 mmol) and 50% solution of T3P in ethyl acetate at RT. The mixture was stirred at RT for 16 h, diluted with water (2 mL) and extracted with EtOAc (20 mL). The organic layer was washed with brine (5 mL) and concentrated. The crude residue was purified on HPLC RP C18 column with gradient MeCN/H_2O (5% to 95%) to give **13** (0.021 g, 0.016 mmol, 42%) as colourless powder.
^{1}H NMR (500 MHz, $CDCl_3$, mixture of rotamers, only peaks of the major rotamer are reported): δ = 7.42-7.37 (m, 1H); 7.36 (d, J=8.6Hz, 2H); 7.32 (d, J-8.6Hz, 2H); 7.22 (dt, J=1.7Hz, J=7.8Hz, 1H); 7.10-6.99 (m, 1H); 6.89 (t, J=7.6Hz,1H); 6.86-6.76 (m, 2H); 6.73-6.69 (m, 1H); 6.68 (s, 1H); 6.52-6.41 (m, 2H); 6.49 (s, 1H); 6.17 (dd, J=1.8Hz, J=6.0Hz, 1H); 5.51 (d, J=4.6Hz, 1H); 4.67-4.56 (m, 1H); 4.50 (ABq, J=14.8Hz, 2H); 4.47-4.34 (m, 2H); 4.27 (d, J=10.8Hz, 1H); 4.00 (ddd, J=3.6Hz, 1H); 3.88-3.84 (m, 12H); 3.80 (s, 3H); 3.70 (s, 3H); 3.69-3.64 (m, 2H); 3.62-3.51 (m, 11H); 3.50-3.40 (m, 2H); 2.68 (s, 3H); 2.69-2.46 (m, 2H); 2.43 (s, 3H); 2.33-1.93 (m, 6H); 1.82-1.73 (m, 1H); 1.72-1.64 (m, 1H); 1.70 (s, 3H); 1.05 (t, J=7.4Hz, 3H); 0.90 (t, J=7.2Hz, 3H).
$C_{67}H_{84}ClN_6O_{15}S$, expected for $[M+H]^+$ 1279.54, found $[M+H]^+$ 1279.6

HDPIC was synthesized by Natalia Shpiro and is detailed within co-submitted manuscript (Brewer et al, joint submission).

References

1. Nowak, R.P., Xiong, Y., Kirmani, N., Kalabathula, J., Donovan, K.A., Eleuteri, N.A., Yuan, J.C., and Fischer, E.S. (2021). Structure-Guided Design of a "Bump-and-Hole" Bromodomain-Based Degradation Tag. Journal of Medicinal Chemistry *64*, 11637-11650. 10.1021/acs.jmedchem.1c00958.
2. Nakanishi, M., Tahara, T., Araki, K., Shiroki, M., Tsumagari, T., and Takigawa, Y. (1973). Studies on Psychotropic-Drugs .18. Synthesis and Structure-Activity Relationships of 5-Phenyl-1,3-Dhydro-2h-Thieno[2,3-E] [1,4] Diazepin-2-Ones. Journal of Medicinal Chemistry *16*, 214-219. DOI 10.1021/jm00261a010.
3. Filippakopoulos, P., Qi, J., Picaud, S., Shen, Y., Smith, W.B., Fedorov, O., Morse, E.M., Keates, T., Hickman, T.T., Felletar, I., et al. (2010). Selective inhibition of BET bromodomains. Nature *468*, 1067-1073. 10.1038/nature09504.
4. Simpson, L.M., Glennie, L., Brewer, A., Zhao, J.F., Crooks, J., Shpiro, N., and Sapkota, G.P. (2022). Target protein localization and its impact on PROTAC-mediated degradation. Cell Chemical Biology *29*, 1482-+. 10.1016/j.chembiol.2022.08.004.
